# Synthesis of Novel Symmetrical 1,4-Disubstituted 1,2,3-Bistriazole Derivatives via ‘Click Chemistry’ and Their Biological Evaluation

**DOI:** 10.3390/molecules21050659

**Published:** 2016-05-19

**Authors:** Esra Düğdü, Dilek Ünlüer, Fatih Çelik, Kemal Sancak, Şengül Alpay Karaoğlu, Arzu Özel

**Affiliations:** 1 Department of Chemistry, Karadeniz Technical University, Trabzon 61080, Turkey; dunluer@yahoo.com (D.Ü.); fatih.celik@ktu.edu.tr (F.Ç.); ksancak@ktu.edu.tr (K.S.); 2 Department of Biology, Recep Tayyip Erdoğan University, Rize 53100, Turkey; sengul.karaoglu@erdogan.edu.tr; 3 Faculty of Pharmacy, Karadeniz Technical University, Trabzon 61080, Turkey; arzuozenozel@ktu.edu.tr

**Keywords:** 1,2,3-triazole, 1,3-dipolar cycloaddition, antioxidant activity, antimicrobial activity

## Abstract

A series of symmetric bis-1,2,3-triazole compounds **2**–**5**(**a**–**f**) were synthesized as potential antioxidant agents via click chemistry. Their structures were confirmed by ^1^H-NMR and ^13^C-NMR. All of the synthesized compounds were subjected to antioxidant and antimicrobial assays. The antioxidant activity of these compounds (AChE inhibition, DPPH and SOD activities) was evaluated. Compound **2f** was found to show the highest AChE inhibition activity of all compounds, while compound **3b** showed a strong inhibitory effect on DPPH radical and compound **2a** was the most effective of all compounds for SOD activity. All synthesized compounds were found to possess moderate antibacterial activity against the bacteria *E. coli* and *Y*.*pseudotuberculosis*.

## 1. Introduction

**Figure 1:**
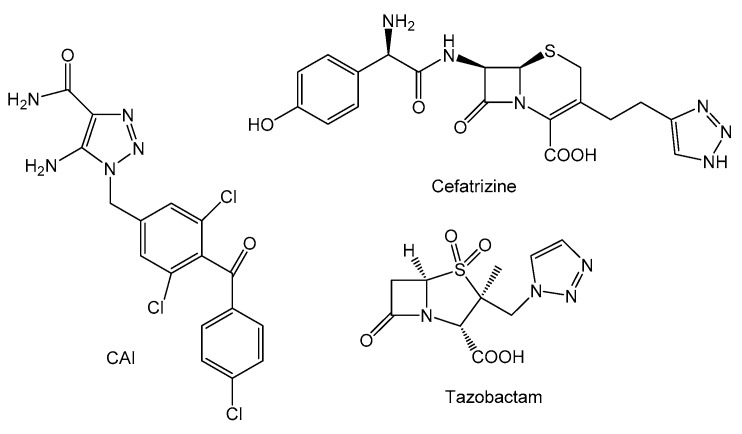
Potential pharmaceuticals based on 1,2,3-triazoles.

*N*-heterocyclic compounds, especially 1,2,3-triazoles, have received special attention because of their widespread use in the synthetic and medicinal chemistry. 1,2,3-Triazoles are considered as interesting heterocycles due to their important pharmacological activities such as antiprotozoal [1], anticonvulsant, anti-HIV [2,3], antimicrobial, antioxidant [4], antifungal [5,6,7], antimalarial [8], anticancer [9], antiinflammatory, and antitubercular agents [10,11], *etc*. There are however very few 1,2,3-triazole-containing molecules on the market or in the last stage of clinical trials. Potential pharmaceuticals based on 1,2,3-triazoles include the anticancer compound carboxy amido triazole (CAI), the β-lactam antibiotic tazobactum, the cephalosporin cefatrizine, and so on (Figure 1) [12].

Substituted 1,2,3-triazole derivatives have been considered as vital structural functionality units commonly available as versatile building blocks in natural products, dyes, corrosion inhibitors, photo stabilizers, photographic materials and agrochemicals [13,14].

The copper-catalyzed azide-alkyne cycloaddition reaction is known for its high fidelity in the presence of many functional groups and under demanding reaction conditions. The experimental simplicity and high selectivity of this process have been exploited in many applications in synthetic and medicinal chemistry, bioconjugations, materials science and polymer chemistry [15]. The copper(I)-catalyzed reaction sequence which regiospecifically joins azides and terminal acetylenes gives only 1,4-disubstituted 1,2,3-triazoles [16]. The process is experimentally simple and appears to have enormous scope.

Here we report an efficient synthesis of novel bis-1,2,3-triazole derivatives **2**–**5**(**a**–**f**) containing alkyl chain bridges via copper-catalyzed click chemistry. In connection with our ongoing programme on the synthesis of bistriazole compounds we were interested in exploring the above cycloaddition reaction where some of the key building blocks contained substituted phenoxy groups. Moreover, we evaluated antioxidant and antimicrobial activity of the products against various microorganisms as 1,2,3-triazole compounds are known to have significant biological activity.

## 2. Results and Discussion

**Scheme 1:**
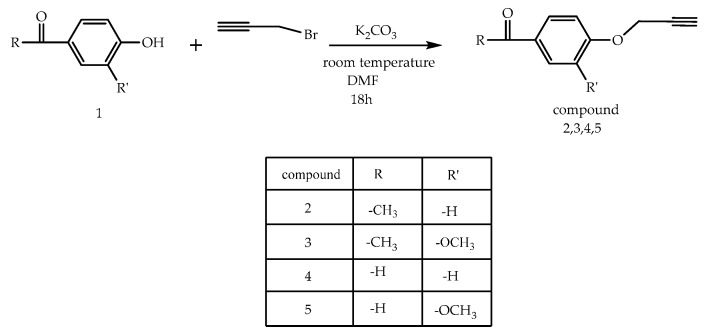

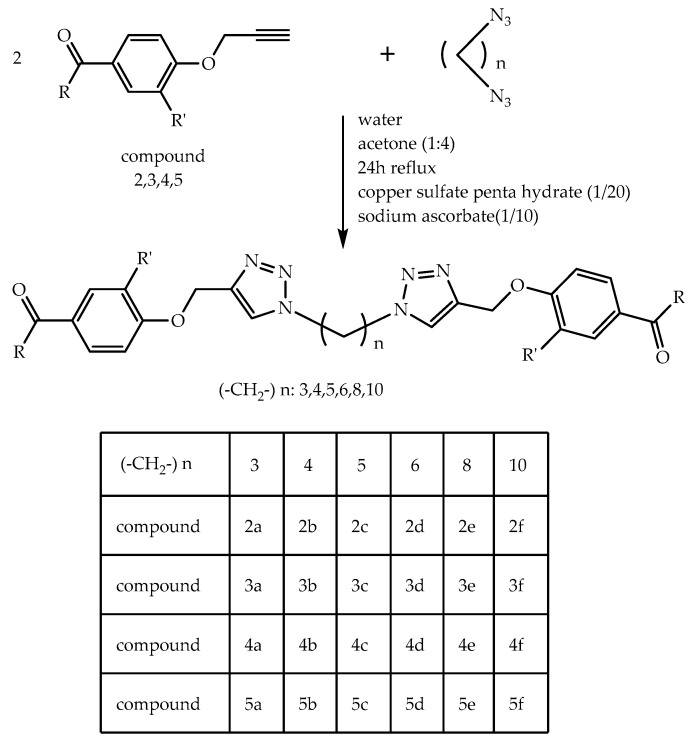
Synthetic pathway for the preparation of compounds **2**–**5**(**a**–**f**).

The synthesis of the intermediate and target compounds was performed according to the reactions outlined in Scheme 1. As shown, commercially available hydroxy acetophenones or benzaldehydes were subjected to O-alkylation with propargyl bromide in the presence of K_2_CO_3_ in DMF. The starting compounds **2**–**5** were obtained following a previously reported literature procedure [17]. Then compounds **2**–**5** were reacted with the bisalkylazide derivatives 1,3-diazido-propane, 1,4-diazidobutane, 1,5-diazidopentane, 1,6-diazidohexane, 1,8-diazidooctane and 1,10-diazidodecane ((-CH_2_-)n, n = 3, 4, 5, 6, 8, 10) under standard “click chemistry” conditions using a copper sulfate pentahydrate/sodium ascorbate system as catalyst, also known as the Huisgen 1,3-dipolar cycloaddition reaction. The reaction conditions in this step are crucial. Because when *tert*-butyl alcohol/water or ethanol/water solutions were used as solvent this reaction failed to afford the target molecules under these conditions, it was tested with a (1:4) ratio acetone/water solvent system whereby unlike in those solvent systems, the synthesis of the target molecules **2**–**5**(**a**–**f**)) was accomplished.

The analytical and spectroscopic data of compounds **2**–**5** confirmed this by the additional signals derived from the –CH_2_C≡CH groups at the expected chemical shift values. Moreover, compounds **2**–**5** gave a stable [M]^+^ ion peak. The ^1^H-NMR spectra of compounds **2**–**5**(**a**–**f**) displayed no signals belonging to the –CH_2_C≡CH group; instead, new signals derived from the 1,2,3-triazole structure appeared at 8.13–8.45 ppm (-trz CH) integrating for two protons. Furthermore, compounds **2**–**5**(**a**–**f**) gave relatively stable [M]^+^ ion peaks.

The IR spectra of compounds **2**–**5**(**a**–**f**) displayed a new sharp signal at 1600 cm^−1^ corresponding C=C absorptions, while the acetylene peak in **2**–**5** disappeared. Also different from compounds **2**–**5** the ^1^H-NMR spectra of the target compounds exhibited additional signals due to the 1,2,3-triazole moiety which were observed at 8.13–8.45 ppm, the expected chemical shift values. Moreover, the N–CH_2_ and –CH_2_ proton signals belonging to compounds **2**–**5**(**a**–**f**) were observed in the 4.33–4.93 ppm range. In the ^13^C-NMR spectra of the target compounds the acetylene carbon peaks observed in the 70–80 ppm range disappeared with the formation of 1,2,3-triazole rings, and quaternary carbon peaks were observed in the range of 140–145 ppm. In addition, compounds **2**–**5**(**a**–**f**) gave relatively stable molecular ion peaks in the corresponding mass spectra.

The biological activity results of all the compounds were expressed as IC_50_ values and are presented as mean ± S.D. of three independent experiments in Table 1. All of the compounds displayed promising AChE inhibitory activities, with IC_50_ values comparable to galantamine used as the reference drug. As can be seen in Table 1, the IC_50_ values of all the compounds examined were in the 50.80–88.60 µM range. The IC_50_ values of the compounds of series **2**, **2a**–**2f** indicated remarkable inhibitory activities but all of the rest remained below 70 μM. Compound **2f** (50.80 (±1.01) µM) displayed the highest inhibition activity of all compounds. The IC_50_ values of the compounds of series **3**, **3a**–**3f** were below 80 μM. In this series compound **3b** displayed much higher activity than the other compounds. Compound **4d** showed important activity in the compound **4** series, while compound **5e** displayed promising inhibitory activity in the **5** series.

Some 1,2,3-triazole compounds are known to act either as inhibitors of free radical production or as radical scavengers. Therefore, the potential antioxidant property of the new 1,2,3-trizole compounds was evaluated by determining their DPPH and SOD radical scavenging ability. DPPH is a stable free radical that accepts an electron or hydrogen radical to become a stable species [18]. Due to its odd electron, DPPH gives a strong absorption band at 517 nm. Antioxidants are suitable reducing agents and neutralize the free radicals by pairing the DPPH odd electron with a hydrogen atom, and the DPPH solution turns yellow, observed as the decrease in absorbance at 517 nm [19].

**Figure 2:**
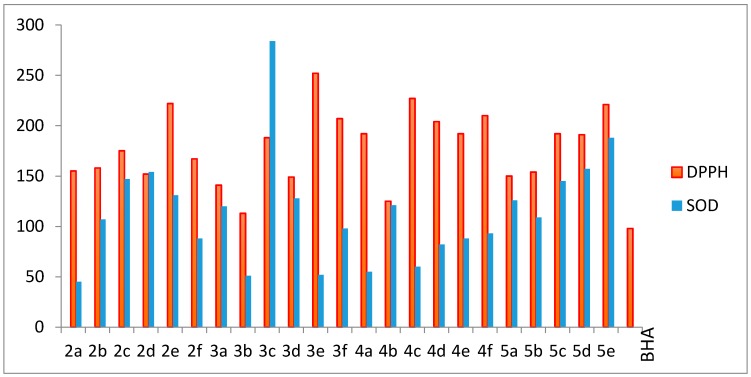
Graphical representation of the antioxidant activity of the tested compounds.

In our study, we evaluated the DPPH scavenging activity of compounds and a standard antioxidant such as BHA as IC_50_ values (Table 1 and Figure 2). Compound **3b** with an IC_50_ value of 113.63 µg/mL showed a strong inhibitory effect on DPPH radical. All other compounds showed moderate activities compared to BHA (IC_50_ 98.56 µg/mL).

The SOD activity of the compounds was investigated by an NBT assay and the results are shown in Table 1 and Figure 2. Compound **2a** (IC_50_ = 45.12 µg/mL) was the most effective of all compounds for scavenging superoxide radicals (O_2_^−•^). Compounds **3b** (IC_50_ = 51.37 µg/mL), **3e** (IC_50_ = 52.89 µg/mL) and **4a** (IC_50_ = 55.67 µg/mL) also exhibited strong superoxide radical scavenging activity.

All the synthesized compounds were found to possess antibacterial activity against the bacteria *E*. *coli* and *Y*. *pseudotuberculosis*, which are Gram-negative enteric (intestinal origin) species. In some molecules, antimycotic effects were observed at very low levels against *S*. *cerevisiae* and *C*. *albicans* yeast. None of the synthesized molecules were observed to have antibacterial activity against Gram negative bacteria (*P*. *aeruginosa*), other Gram positive bacteria (*B. cereus, S. aureus* and *E. faecalis*) or antitubercular activity against M. *smegmatis*.

## 3. Experimental Section

### 3.1. General Information

Melting points were measured on an Electrothermal apparatus and are uncorrected. ^1^H-NMR and ^13^C-NMR spectra were recorded on an FT-400 NMR spectrophotometer (Agilent, Santa Clara, CA, USA) in DMSO-*d*_6_. IR spectra were recorded on a Spectrum one FT-IR spectrometer (Perkin-Elmer, Waltham, MA, USA) in KBr pellets. The MS spectra were measured with a Micromass Quattro LC/ULTIMA LC-MS/MS spectrometer (Waters, Milford, MA, USA) with EtOH as solvent. The experiment was performed in the positive ion mode. Elemental analyses were performed on a Hewlett-Packard 185 CHN analyzer (Avondale, PA, USA). All the chemicals were obtained from Fluka Chemie AG (Buchs, Switzerland) and Sigma-Aldrich (St. Louis, MO, USA). Electric eel acetyl-cholinesterase enzyme (AchE, Type-VI-S; EC 3.1.1.7), acetylthiocholine iodide (ATCI), 5,5-dithiobis(2-nitrobenzoic) acid (DTNB), galantamine and 2,2-diphenyl-1-picrylhydrazyl (DPPH), methanol, and Trisma-base were purchased from Sigma-Aldrich and used for the measurement of the acetyl-cholinesterase inhibition and antioxidant activities.

### 3.2. Synthesis of Compounds ***2**–**5***

Acetylene derivatives **1** (0.010 mol) and potassium carbonate (0.015 mol) were refluxed in DMF for 1 h. The reaction mixture was cooled to room temperature and propargyl bromide (0.01 mol) was added and the mixture stirred at room temperature for 24 h. The reaction mixture was poured into 200 mL of ice-cold water. The solid obtained was washed with water and recrystallized from ethanol to afford the desired title compounds **2**–**5** [17].

### 3.3. General Synthesis Method for the ***2**–**5**(**a**–**f**)*

Bisalkylazide derivatives (-CH_2_-)_n_ (n = 3,4,5,6,8,10) (0.01 mol) and compounds **2**–**5** (0.02 mol) were stirred in (1:4) water/acetone solution at room temperature for one hour. Then copper sulfate pentahydrate (1/20) and sodium ascorbate (1/10) were added and the reaction mixture was refluxed for 24 h. At the end of the reaction the flask contents were poured into ice water and a solid was obtained, which was filtered, washed with water and recrystallized from DMF-ethanol to afford the target compounds **2**–**5**(**a**–**f**).

*1,1′-((((Propane-1,3-diylbis(1H-1,2,3-triazole-1,4-diyl))bis(methylene))bis(oxy))bis(4,1-phenylene))bis(ethan-1-one)* (**2a**). Yield: 80.02%; m.p. 181–182°C; IR (ν,cm^−1^): 1674 (C=O), 1605 (C=C), 1255 (C-O); ^1^H-NMR (δ ppm): 2.46 (m, 2H, -CH_2_-), 2.51 (s, 6H, -CH_3_), 4.43 (t, 4H, N-CH_2_), 5.25 (s, 2H, O-CH_2_), 7.13–7.15 (d, 4H, Arom. H), 7.92–7.94 (d, 4H, Arom. H), 8.30 (s, 2H, Arom. -1,2,3-trz. H); ^13^C-NMR (δ ppm): 26.85 (-CH_3_), 30.61 (-CH_2_-), 47.25 (N-CH_2_), 61.85 (O-CH_2_), Arom. C [115.02 (CH), 125.30(trz. CH), 130.64(C), 130.91 (CH), 142.76 (trz. C-N), 162.31 (C)], 196.76 (C=O); Anal. Calcd (%) for C_25_H_26_N_6_O_4_ (474.20): C, 63.28; H, 5.52; N, 17.71; found: C, 63.22; H, 5.56; N, 17.73; MS (ESI): *m*/*z* (%) 475.55 [M + 1]^+^.

*1,1′-((((Butane-1,4-diylbis(1H-1,2,3-triazole-1,4-diyl))bis(methylene))bis(oxy))bis(4,1-phenylene))bis(ethan-1-one)* (**2b**). Yield: 75.02%; m.p. 183–184 °C; IR (ν, cm^−1^): 1677 (C=O), 1600 (C=C),1248 (C-O); ^1^H-NMR (δ ppm): 1.78 (bs, 4H, -CH_2_-), 2.49 (s, 6H, -CH_3_), 4.39 (bs, 4H, N-CH_2_), 5.22 (s, 4H, O-CH_2_), 7.10–7.13 (d, 4H, Arom. H), 7.89–7.92 (d, 4H, Arom. H), 8.23 (s, 2H, Arom. -1,2,3-trz. H); ^13^C-NMR (δ ppm): 26.07 (-CH_3_), 27.15 (-CH_2_-), 49.10 (N-CH_2_), 63.92 (O-CH_2_), Arom. C [114.94 (CH), 128.54 (trz. CH), 130.51 (C), 130.91 (CH), 142.58 (trz. C-N), 162.38 (C)], 196.76 (C=O); Anal. Calcd (%) for C_26_H_28_N_6_O_4_ (488.22): C, 63.92; H, 5.78; N, 17.20; found: C, 63.91; H, 5.75; N, 17.21; MS (ESI): *m*/*z* (%) 527.23 [M + 1]^+^.

*1,1′-((((Pentane-1,5-diylbis(1H-1,2,3-triazole-1,4-diyl))bis(methylene))bis(oxy))bis(4,1-phenylene))bis(ethan-1-one)* (**2c**). Yield: 75.02%; m.p. 147–148 °C; IR (ν, cm^−1^): 1673 (C=O), 1604 (C=C), 1246 (C-O); ^1^H-NMR (δ ppm): 1.20–1.30 (m, 2H, -CH_2_-), 1.80–1.85 (m, 4H, -CH_2_-), 2.48 (s, 6H, -CH_3_), 4.33 (bs, 4H, N-CH_2_), 5.21 (s, 4H, O-CH_2_), 7.10–7.13 (d, 4H, Arom. H), 7.89–7.92 (d, 4H, Arom. H), 8.22 (s, 2H, Arom. -1,2,3-trz. H); ^13^C-NMR (δ ppm) : 23.20 (-CH_2_-), 26.83 (-CH_3_), 29.43 (-CH_2_-), 49.59 (N-CH_2_), 61.84 (O-CH_2_), Arom. C [114.96 (CH), 125.10(trz.CH), 130.56 (C), 130.89 (CH), 141.35 (trz. C-N), 162.28 (C)], 196.74 (C=O); Anal. Calcd (%) for C_27_H_30_N_6_O_4_ (502.23): C, 64.53; H, 6.02; N, 16.72; found: C, 64.50; H, 6.01; N, 16.75; MS (ESI): *m*/*z* (%) 503.33 [M + 1]^+^.

*1,1′-((((Hexane-1,6-diylbis(1H-1,2,3-triazole-1,4-diyl))bis(methylene))bis(oxy))bis(4,1-phenylene))bis(ethan-1-one)* (**2d**). Yield: 78.15%; m.p. 166–167 °C; IR (ν, cm^−1^): 1677 (C=O), 1600 (C=C), 1246 (C-O); ^1^H-NMR (δ ppm): 1.15–1.25 (m, 4H, -CH_2_-), 1.71–1.82 (m, 4H, -CH_2_-), 2.47 (s, 6H, -CH_3_), 4.30 (bs, 4H, N-CH_2_), 5.20 (s, 4H, O-CH_2_), 7.10–7.14 (d, 4H, Arom. H), 7.87–7.92 (d, 4H, Arom. H), 8.21 (s, 2H, Arom. -1,2,3-trz. H); ^13^C-NMR (δ ppm): 25.63 (-CH_2_-) 26.82 (-CH_3_), 29.87 (-CH_2_-), 49.71 (N-CH_2_), 61.84 (O-CH_2_), Arom. C [114.98 (CH), 128.45(trz. CH), 125.48 (C), 130.88 (CH), 142.55 (trz. C-N), 165.40 (C)], 196.95 (C=O); Anal. Calcd (%) for C_28_H_32_N_6_O_4_ (516.25): C, 65.10; H, 6.24; N, 16.27; found: C, 65.12; H, 6.25; N, 16.25; MS (ESI): *m*/*z* (%) 517.47 [M + 1]^+^.

*1,1′-((((Octane-1,8-diylbis(1H-1,2,3-triazole-1,4-diyl))bis(methylene))bis(oxy))bis(4,1-phenylene))bis(ethan-1-one)* (**2e**). Yield: 76.15%; m.p. 169–170 °C; IR (ν, cm^−1^): 1678 (C=O), 1601 (C=C), 1252 (C-O); ^1^H-NMR (δ ppm) : 1.16–1.24 (m, 8H, -CH_2_-), 1.72–1.81 (m, 4H, -CH_2_-), 2.48 (s, 6H, -CH_3_), 4.33 (bs, 4H, N-CH_2_), 5.21 (s, 4H, O-CH_2_), 7.10–7.12 (d, 4H, Arom. H), 7.89–7.91 (d, 4H, Arom. H), 8.22 (s, 2H, Arom. -1,2,3-trz. H); ^13^C-NMR (δ ppm): 26.13 (-CH_2_-), 26.83 (-CH_2_-), 28.59 (-CH_3_), 30.04 (-CH_2_-), 49.81 (N-CH_2_), 61.83 (O-CH_2_), Arom. C [114.99 (CH), 125.01 (trz. CH), 130.55 (C), 130.87 (CH), 142.38 (trz. C-N), 162.28 (C)], 196.72 (C=O); Anal. Calcd (%) for C_30_H_36_N_6_O_4_ (544.28): C, 66.16; H, 6.66; N, 15.43; found: C, 66.18; H, 6.64; N, 15.44; MS (ESI): *m*/*z* (%) 545.14 [M + 1]^+^.

*1,1′-((((Decane-1,10-diylbis(1H-1,2,3-triazole-1,4-diyl))bis(methylene))bis(oxy))bis(4,1-phenylene))bis-(ethan-1-one)* (**2f**). Yield: 74.15%; m.p. 165–166 °C; IR (ν, cm^−1^): 1677 (C=O), 1603 (C=C), 1251 (C-O); ^1^H-NMR (δ ppm): 1.21–1.22 (m, 12H, -CH_2_-), 1.81 (m, 4H, -CH_2_-), 2.51 (s, 6H, -CH_3_), 4.35 (bs, 4H, N-CH_2_), 5.25 (s, 4H, O-CH_2_), 7.12–7.14 (d, 4H, Arom. H), 7.91–7.93 (d, 4H, Arom. H), 8.20 (s, 2H, Arom. -1,2,3-trz. H); ^13^C-NMR (δ ppm): 24.48 (-CH_2_-), 25.26 (-CH_2_-), 27.20 (-CH_2_-), 27.92 (-CH_3_), 30.78 (-CH_2_-), 50.42 (N-CH_2_), 62.25 (O-CH_2_), Arom. C [115.67 (CH), 128.42 (trz. CH), 130.30 (C), 130.69 (CH), 142.67 (trz. C-N), 162.54 (C)], 197.65 (C=O); Anal. Calcd (%) for C_32_H_40_N_6_O_4_ (572.31): C, 67.11; H, 7.04; N, 14.67; found: C, 67.13; H, 7.06; N, 14.64; MS (ESI): *m*/*z* (%) 595.35 [M + Na]^+^.

*1-(4-((1-(3-(4-((4-Acetyl-2-methoxyphenoxy)methyl)-1H-1,2,3-triazol-1-yl)propyl)-1H-1,2,3-triazol-4-yl)-methoxy)-2-methoxyphenyl)ethan-1-one* (**3a**). Yield: 78.15%; m.p. 157–159 °C; IR (ν, cm^−1^): 1674 (C=O), 1587 (C=C), 1267 (C-O); ^1^H-NMR (δ ppm): 2.35–2.45 (m, 8H,(-CH_2 _ and -CH_3_)), 3.79 (s, 6H, -OCH_3_), 4.42 (t, 4H, N-CH_2_), 5.23 (s, 4H, O-CH_2_), 7.26–7.29 (d, 2H, Arom. H), 7.44 (s, 2H, Arom. H), 7.61–7.63 (d, 2H, Arom. H), 8.30 (s, 2H, Arom. -1,2,3-trz. CH); ^13^C-NMR (δ ppm): 26.79 (-CH_3_), 30.64 (-CH_2_-), 47.26 (N-CH_2_), 55.94 (-OCH_3_), 62.17 (O-CH_2_), Arom. C [110.93 (CH), 112.81 (CH), 123.34 (CH), 125.45 (trz. CH), 130.73 (C) ,142.71 (trz. C-N), 149.19 (C), 152.16 (C)], 196.85 (C=O); Anal. Calcd (%) for C_27_H_30_N_6_O_6_ (534.22): C, 60.66; H, 5.66; N, 15.72; found: C, 60.61; H, 5.63; N, 15.70; MS (ESI): *m*/*z* (%) 557.35 [M + Na]^+^.

*1-(4-((1-(4-(4-((4-Acetyl-2-methoxyphenoxy)methyl)-1H-1,2,3-triazol-1-yl)butyl)-1H-1,2,3-triazol-4-yl)-methoxy)-2-methoxyphenyl)ethan-1-one* (**3b**). Yield: 76.14%; m.p. 158–159 °C; IR (ν, cm^−1^): 1654 (C=O), 1590 (C=C), 1262 (C-O); ^1^H-NMR (δ ppm): 1.78–1.80 (m, 4H, -CH_2_-), 2.48 (s, 6H, -CH_3_), 3.77 (s, 6H, -OCH_3_), 4.39 (t, 4H, N-CH_2_), 5.20 (s, 4H, O-CH_2_), 7.23–7.25 (d, 2H, Arom. H), 7.42 (s, 2H, Arom. H), 7.58–7.60 (d, 2H, Arom. H), 8.22 (s, 2H, Arom. -1,2,3-trz. CH); ^13^C-NMR (δ ppm): 26.92 (-CH_2_-), 30.70 (-CH_3_), 48.25 (N-CH_2_), 55.90 (-OCH_3_), 62.17 (O-CH_2_), Arom. C [110.16 (CH), 113.85 (CH), 123.44 (CH), 125.43 (trz. CH), 130.38 (C) ,142.42 (trz. C-N), 149.74 (C), 153.15 (C)], 196.65 (C=O); Anal. Calcd (%) for C_28_H_32_N_6_O_6_ (548.24): C, 61.30; H, 5.88; N, 15.32; found: C, 61.32; H, 5.86; N, 15.34; MS (ESI): *m*/*z* (%) 571.38 [M + Na]^+^.

*1-(4-((1-(5-(4-((4-Acetyl-2-methoxyphenoxy)methyl)-1H-1,2,3-triazol-1-yl)pentyl)-1H-1,2,3-triazol-4-yl)-methoxy)-2-methoxyphenyl)ethan-1-one* (**3c**). Yield: 74.14%; m.p. 165–166 °C; IR (ν, cm^−1^): 1668 (C=O), 1584 (C=C), 1261 (C-O); ^1^H-NMR (δ ppm): 1.21 (m, 2H, -CH_2_-), 1.84 (m, 4H, -CH_2_-), 2.50 (s, 6H, -CH_3_), 3.77 (s, 6H, -OCH_3_), 4.35 (t, 4H, N-CH_2_), 5.20 (s, 4H, O-CH_2_), 7.22–7.24 (d, 2H, Arom. H), 7.42 (s, 2H, Arom. H), 7.57–7.59 (d, 2H, Arom. H), 8.22 (s, 2H, Arom. -1,2,3-trz. CH); ^13^C-NMR (δ ppm): 23.25 (-CH_2_-), 26.73 (-CH_2_-), 29.49 (-CH_3_), 49.59 (N-CH_2_), 55.84 (-OCH_3_), 62.14 (O-CH_2_), Arom. C [110.75 (CH), 112.61 (CH), 123.33 (CH), 125.21 (trz. CH), 130.62 (C) , 142.53 (trz. C-N), 149.11 (C), 152.12 (C)], 196.82 (C=O); Anal. Calcd (%) for C_29_H_34_N_6_O_6_ (562.25): C, 61.91; H, 6.09; N, 14.94; found; C, 61.92; H, 6.10; N, 14.95; MS (ESI): *m*/*z* (%) 585.35 [M + Na]^+^.

*1-(4-((1-(6-(4-((4-Acetyl-2-methoxyphenoxy)methyl)-1H-1,2,3-triazol-1-yl)hexyl)-1H-1,2,3-triazol-4-yl)-methoxy)-2-methoxyphenyl)ethan-1-onen* (**3d**). Yield: 72.14%; m.p. 205–207 °C; IR (ν, cm^−1^): 1667 (C=O), 1582 (C=C), 1260 (C-O); ^1^H-NMR (δ ppm): 1.19–1.24 (m, 4H, -CH_2_-), 1.76–1.80 (m, 4H, -CH_2_-), 2.50 (s, 6H, -CH_3_), 3.76 (s, 6H, -OCH_3_), 4.32 (t, 4H, N-CH_2_), 5.20 (s, 4H, O-CH_2_), 7.23–7.25 (d, 2H, Arom. H), 7.42 (s, 2H, Arom. H), 7.58–7.60 (d, 2H, Arom. H), 8.23 (s, 2H, Arom. -1,2,3-trz. CH); ^13^C-NMR (δ ppm) δ : 25.66 (-CH_2_-), 26.76 (-CH_2_-), 29.91 (-CH_3_), 49.72 (N-CH_2_), 55.85 (-OCH_3_), 62.13 (O-CH_2_), Arom. C [110.78 (CH), 112.65 (CH), 123.33 (CH), 125.19 (trz. CH), 130.62 (C) , 142.50 (trz. C-N), 149.12 (C), 152.12 (C)], 196.82 (C=O); Anal. Calcd (%) for C_30_H_36_N_6_O_6_ (576.27): C, 62.49; H, 6.29; N, 14.57; found: C, 62.48; H, 6.30; N, 14.59; MS (ESI): *m*/*z* (%) 599.38 [M + Na]^+^.

*1-(4-((1-(8-(4-((4-Acetyl-2-methoxyphenoxy)methyl)-1H-1,2,3-triazol-1-yl)octyl)-1H-1,2,3-triazol-4-yl)methoxy)-2-methoxyphenyl)ethan-1-one* (**3e**). Yield: 75.14%; m.p. 172–174 °C; IR (ν, cm^−1^): 1669 (C=O), 1585 (C=C), 1266 (C-O); ^1^H-NMR (δ ppm): 1.18–1.22 (m, 8H, -CH_2_-), 1.75–1.80 (m, 4H, -CH_2_-), 2.50 (s, 6H, -CH_3_), 3.76 (s, 6H, -OCH_3_), 4.33 (t, 4H, N-CH_2_), 5.21 (s, 4H, O-CH_2_), 7.23–7.25 (d, 2H, Arom. H), 7.42 (s, 2H, Arom. H), 7.58–7.60 (d, 2H, Arom. H), 8.23 (s, 2H, Arom. -1,2,3-trz. CH); ^13^C-NMR (δ ppm) : 26.17 (-CH_2_-), 26.74 (-CH_2_-), 28.60 (-CH_2_-), 30.08 (-CH_3_), 49.80 (N-CH_2_), 55.84 (-OCH_3_), 62.13 (O-CH_2_), Arom. C [110.78 (CH), 112.65 (CH), 123.31 (CH), 125.18 (trz. CH), 130.62 (C), 142.49 (trz. C-N), 149.13 (C), 152.12 (C)], 196.79 (C=O); Anal. Calcd (%) for C_32_H_40_N_6_O_6_ (604.30): C, 63.56; H, 6.67; N, 13.90; found: C, 63.58; H, 6.68; N, 13.92; MS (ESI): *m*/*z* (%) 605.31 [M + 1]^+^.

*1-(4-((1-(10-(4-((4-Acetyl-2-methoxyphenoxy)methyl)-1H-1,2,3-triazol-1-yl)decyl)-1H-1,2,3-triazol-4-yl)-methoxy)-2-methoxyphenyl)ethan-1-one* (**3f**). Yield: 73.35%; m.p. 120–122 °C; IR (ν, cm^−1^): 1670 (C=O), 1587 (C=C), 1266 (C-O); ^1^H-NMR (δ ppm) : 1.04–1.18 (m, 12H, -CH_2_-), 1.75–1.79 (m, 4H, -CH_2_-), 2.50 (s, 6H, -CH_3_), 3.76 (s, 6H, -OCH_3_), 4.33 (t, 4H, N-CH_2_), 5.20 (s, 4H, O-CH_2_), 7.23–7.25 (d, 2H, Arom. H), 7.42 (s, 2H, Arom. H), 7.58–7.60 (d, 2H, Arom. H), 8.23 (s, 2H, Arom. -1,2,3-trz. CH); ^13^C-NMR (δ ppm): 26.26 (-CH_2_-), 26.76 (-CH_2_-), 28.74 (-CH_2_-), 29.16 (-CH_2_-), 30.14 (-CH_3_), 49.83 (N-CH_2_), 55.86 (-OCH_3_), 62.12 (O-CH_2_), Arom. C [110.79 (CH), 112.67 (CH), 123.32 (CH), 125.19 (trz. CH), 130.62 (C), 142.50 (trz. C-N), 149.13 (C), 152.12 (C)], 196.79 (C=O); Anal. Calcd (%) for C_34_H_44_N_6_O_6_ (632.33): C, 64.54; H, 7.01; N, 13.28; found: C, 64.56; H, 7.04; N, 13.29; MS (ESI): *m*/*z* (%) 655.24 [M + Na]^+^.

*4,4′-(((Propane-1,3-diylbis(1H-1,2,3-triazole-1,4-diyl))bis(methylene))bis(oxy))dibenzaldehyde* (**4a**). Yield: 70.18%; m.p. 115–116 °C; IR (ν, cm^−1^): 1688 (C=O), 1598 (C=C), 1253 (C-O); ^1^H-NMR (δ ppm) : 2.43–2.45 (m, 2H, -CH_2_-), 4.43 (t, 4H, N-CH_2_), 5.27 (s, 4H, O-CH_2_), 7.21–7.23 (d, 4H, Arom. H), 7.84–7.86 (d, 4H, Arom. H), 8.30 (s, 2H, Arom. -1,2,3-trz. H), 9.85 (s, 2H, aldehyde-CH); ^13^C-NMR (δ ppm) : 30.61 (-CH_2_-), 47.26 (N-CH_2_), 61.95 (O-CH_2_), Arom. C [115.62 (CH), 125.43 (trz. CH), 130.31 (C), 132.22 (CH), 143.53 (trz. C-N), 163.38 (C)], 191.74 (aldehyde-CH); Anal. Calcd (%) for C_23_H_22_N_6_O_4_ (446.17): C, 61.88; H, 4.97; N, 18.82; found: C, 61.86; H, 4.98; N, 18.84; MS (ESI): *m*/*z* (%) 469.29 [M + Na]^+^.

*4,4′-(((Butane-1,4-diylbis(1H-1,2,3-triazole-1,4-diyl))bis(methylene))bis(oxy))dibenzaldehyde* (**4b**). Yield: 73.53%; m.p. 154–155 °C; IR (ν, cm^−1^): 1682 (C=O), 1601 (C=C), 1245 (C-O); ^1^H-NMR (δ ppm): 1.79 (bs, 4H, -CH_2_-), 4.40 (t, 4H, N-CH_2_), 5.25 (s, 4H, O-CH_2_), 7.20 (s, 4H, Arom. H), 7.84 (s, 4H, Arom. H), 8.24 (s, 2H, Arom. -1,2,3-trz. H), 9.85 (s, 2H, aldehyde-CH); ^13^C-NMR (δ ppm): 27.42 (-CH_2_-), 49.20 (N-CH_2_), 61.95 (O-CH_2_), Arom. C [115.64 (CH), 125.21 (trz. CH), 130.31 (C), 132.22(CH), 142.49 (trz. C-N), 163.39 (C)], 191.77 (aldehyde-CH); Anal. Calcd (%) for C_24_H_24_N_6_O_4_ (460.19): C, 62.60; H, 5.25; N, 18.25; found: C, 62.62; H, 5.26; N, 18.27; MS (ESI): *m*/*z* (%) 483.28 [M + Na]^+^.

*4,4′-(((Pentane-1,5-diylbis(1H-1,2,3-triazole-1,4-diyl))bis(methylene))bis(oxy))dibenzaldehyde* (**4c**). Yield: 76.73%; m.p. 98–99 °C; IR (ν, cm^−1^): 1693 (C=O), 1600 (C=C), 1243 (C-O); ^1^H-NMR (δ ppm) : 1.19–1.21 (m, 2H, -CH_2_-), 1.81–1.84 (m, 4H, -CH_2_-), 4.34 (t, 4H, N-CH_2_), 5.25 (s, 4H, O-CH_2_), 7.20–7.22 (d, 4H, Arom. H), 7.84–7.86 (d, 4H, Arom. H), 8.24 (s, 2H, Arom. -1,2,3-trz. H), 9.85 (s, 2H, aldehyde-CH); ^13^C-NMR (δ ppm): 23.21 (-CH_2_-), 29.45 (-CH_2_-), 49.59 (N-CH_2_), 61.97 (O-CH_2_), Arom. C [115.64 (CH), 125.12 (trz. CH), 130.30 (C), 132.22 (CH), 142.41 (trz. C-N), 163.39 (C)], 191.76 (aldehyde-CH); Anal. Calcd (%) for C_25_H_26_N_6_O_4_ (474.20): C, 63.28; H, 5.52; N, 17.71; found: C, 63.26; H, 5.53; N, 17.73; MS (ESI): *m*/*z* (%) 475.42 [M + 1]^+^.

*4,4′-(((Hexane-1,6-diylbis(1H-1,2,3-triazole-1,4-diyl))bis(methylene))bis(oxy))dibenzaldehyde* (**4d**). Yield: 76.73%; m.p. 100–101 °C; IR (ν, cm^−1^): 1683 (C=O), 1608 (C=C), 1242 (C-O); ^1^H-NMR (δ ppm) : 1.21 (bs, 4H, -CH_2_-), 1.76 (bs, 4H, -CH_2_-), 4.32 (t, 4H, N-CH_2_), 5.26 (s, 4H, O-CH_2_), 7.19–7.21 (d, 4H, Arom. H), 7.83–7.85 (d, 4H, Arom. H), 8.26 (s, 2H, Arom. -1,2,3-trz. H), 9.84 (s, 2H, aldehyde-CH); ^13^C-NMR (δ ppm) : 25.63 (-CH_2_-), 29.87 (-CH_2_-), 49.76 (N-CH_2_), 62.00 (O-CH_2_), Arom. C [115.62 (CH), 130.29 (trz. CH), 132.18 (C) , 132.23 (CH), 142.50 (trz. C-N), 163.38 (C)], 191.71 (aldehyde-CH); Anal. Calcd (%) for C_26_H_28_N_6_O_4_ (488.22): C, 63.92; H, 5.78; N, 17.20; found: C, 63.94; H, 5.77; N, 17.22; MS (ESI): *m*/*z* (%) 489.25 [M + 1]^+^.

*4,4′-(((Octane-1,8-diylbis(1H-1,2,3-triazole-1,4-diyl))bis(methylene))bis(oxy))dibenzaldehyde* (**4e**). Yield: 76.73%; m.p. 94–95 °C; IR (ν, cm^−1^): 1690 (C=O), 1605 (C=C), 1246 (C-O); ^1^H-NMR (δ ppm) : 1.17 (bs, 8H, -CH_2_-), 1.76 (bs, 4H, -CH_2_-), 4.33 (t, 4H, N-CH_2_), 5.26 (s, 4H, O-CH_2_), 7.19–7.21(d, 4H, Arom. H), 7.83–7.85 (d, 4H, Arom. H), 8.25 (s, 2H, Arom. -1,2,3-trz. H), 9.85 (s, 2H, aldehyde-CH); ^13^C-NMR (δ ppm): 26.15 (-CH_2_-), 28.59 (-CH_2_-), 30.06 (-CH_2_-), 49.85 (N-CH_2_), 61.98 (O-CH_2_), Arom. C [115.62 (CH), 125.11 (trz. CH), 130.29 (C), 132.19 (CH), 142.47 (trz. C-N), 163.39 (C)], 191.66 (aldehyde-CH); Anal.Calcd (%) for C_28_H_32_N_6_O_4_ (516.25): C, 65.10; H, 6.24; N, 16.27; found: C, 65.12; H, 6.25; N, 16.28; MS (ESI): *m*/*z* (%) 517.14 [M + 1]^+^.

*4,4′-(((Decane-1,10-diylbis(1H-1,2,3-triazole-1,4-diyl))bis(methylene))bis(oxy))dibenzaldehyde* (**4f**). Yield: 74.16%; m.p. 72–73 °C; IR (ν, cm^−1^): 1688 (C=O), 1604 (C=C), 1250 (C-O); ^1^H-NMR (δ ppm): 1.36 (bs, 6H, -CH_2_-), 1.95 (bs, 4H, -CH_2_-), 4.51 (t, 4H, N-CH_2_), 5.43 (s, 4H, O-CH_2_), 7.38–7.40 (d, 4H, Arom. H), 8.02–8.04 (d, 4H, Arom. H), 8.43 (s, 2H, Arom. -1,2,3-trz. H), 10.03 (s, 2H, aldehyde-CH); ^13^C NMR (δ ppm): 26.22 (-CH_2_-), 28.72 (-CH_2_-), 29.14 (-CH_2_-), 30.09 (-CH_2_-), 49.85 (N-CH_2_), 61.97 (O-CH_2_), Arom. C [115.65 (CH), 125.11 (trz. CH), 130.28 (C), 132.19 (CH), 142.55 (trz. C-N), 163.39 (C)], 191.71 (aldehyde-CH); Anal. Calcd (%) for C_30_H_36_N_6_O_4_ (544.28): C, 66.16; H, 6.66; N, 15.43; found: C, 66.18; H, 6.68; N, 15.44; MS (ESI): *m*/*z* (%) 545.26 [M + 1]^+^.

*4-((1-(3-(4-((4-Formyl-2-methoxyphenoxy)methyl)-1H-1,2,3-triazol-1-yl)propyl)-1H-1,2,3-triazol-4-yl)-methoxy)-2-methoxybenzaldehyde* (**5a**). Yield: 82.69%; m.p. 90–92 °C; IR (ν, cm^−1^): 1676 (C=O), 1588 (C=C), 1261 (C-O); ^1^H-NMR (δ ppm): 2.42–2.44 (m, 2H, -CH_2_-), 3.78 (s, 6H, -OCH_3_), 4.43 (t, 4H, N-CH_2_), 5.25 (s, 4H, O-CH_2_), 7.36 (s, 2H, Arom. H), 7.37–7.39 (d, 2H, Arom. H), 7.52–7.54 (d, 2H, Arom. H), 8.29 (s, 2H, Arom. -1,2,3-trz. H), 9.82 (s, 2H, aldehyde-CH); ^13^C-NMR (δ ppm): 30.63 (-CH_2_-), 47.25 (N-CH_2_), 55.90 (-OCH_3_), 62.19 (O-CH_2_), Arom. C [110.12 (CH), 113.04 (CH), 125.56 (trz. CH), 126.28 (CH), 130.37 (C) ,142.50 (C), 149.73 (trz. C-N), 153.25 (C)], 191.86 (aldehyde-CH); Anal. Calcd (%) for C_25_H_26_N_6_O_6_ (506.19): C, 59.28; H, 5.17; N, 16.59; found: C, 59.27; H, 5.18; N, 16.57; MS (ESI): *m*/*z* (%) 507.25 [M + 1]^+^.

*4-((1-(4-(4-((4-Formyl-2-methoxyphenoxy)methyl)-1H-1,2,3-triazol-1-yl)butyl)-1H-1,2,3-triazol-4-yl)-methoxy)-2-methoxybenzaldehyde* (**5b**). Yield: 79.69%; m.p. 96–97 °C; IR (ν, cm^−1^): 1665 (C=O), 1585 (C=C), 1263 (C-O); ^1^H-NMR (δ ppm) : 1.80 (bs, 4H, -CH_2_-), 3.78 (s, 6H, -OCH_3_), 4.40 (t, 4H, N-CH_2_), 5.24 (s, 4H, O-CH_2_), 7.37 (s, 2H, Arom. H), 7.51 (s, 2H, Arom. H), 7.53 (s, 2H, Arom. H), 8.24 (s, 2H, Arom. -1,2,3-trz. H), 9.82 (s, 2H, aldehyde-CH); ^13^C-NMR (δ ppm): 27.29 (-CH_2_-), 49.20 (N-CH_2_), 55.90 (-OCH_3_), 62.22 (O-CH_2_), Arom. C [110.15 (CH), 113.05 (CH), 125.38 (trz. CH), 126.24 (CH), 130.38 (C), 142.42 (C), 149.74 (trz. C-N), 153.26 (C)], 191.83 (aldehyde-CH); Anal. Calcd (%) for C_26_H_28_N_6_O_6_ (520.21): C, 59.99; H, 5.42; N, 16.14; found: C, 59.98; H, 5.44; N, 16.15; MS (ESI): *m*/*z* (%) 521.23 [M + 1]^+^.

*4-((1-(5-(4-((4-Formyl-2-methoxyphenoxy)methyl)-1H-1,2,3-triazol-1-yl)pentyl)-1H-1,2,3-triazol-4-yl)-methoxy)-2-methoxybenzaldehyde* (**5c**). Yield: 88.60%; m.p. 141–142 °C; IR (ν, cm^−1^): 1675 (C=O), 1586 (C=C), 1261 (C-O); ^1^H-NMR (δ ppm): 1.21 (m, 2H, -CH_2_-), 1.82–1.84 (m, 4H, -CH_2_-), 3.78 (s, 6H, -OCH_3_), 4.34 (t, 4H, N-CH_2_), 5.24 (s, 4H, O-CH_2_), 7.34 (s, 2H, Arom. H), 7.35–7.39 (d, 2H, Arom. H), 7.51–7.54 (d, 2H, Arom. H), 8.23 (s, 2H, Arom. -1,2,3-trz. H), 9.82 (s, 2H, aldehyde-CH); ^13^C-NMR (δ ppm): 23.25 (-CH_2_-), 29.48 (-CH_2_-), 49.60 (N-CH_2_), 55.91 (-OCH_3_), 62.24 (O-CH_2_), Arom. C [110.10 (CH), 113.01 (CH), 125.30 (trz. CH), 126.26 (CH), 130.36 (C), 142.34 (C), 149.73 (trz. C-N), 153.27 (C)], 191.82 (aldehyde-CH); Anal. Calcd (%) for C_27_H_30_N_6_O_6_ (534.22): C, 60.66; H, 5.66; N, 15.72; found: C, 60.68; H, 5.67; N, 15.73; MS (ESI): *m*/*z* (%) 535.36 [M + 1]^+^.

*4-((1-(6-(4-((4-Formyl-2-methoxyphenoxy)methyl)-1H-1,2,3-triazol-1-yl)hexyl)-1H-1,2,3-triazol-4-yl)-methoxy)-2-methoxybenzaldehyde* (**5d**). Yield: 68.60%, m.p. 151–152 °C. IR (ν, cm^−1^): 1681 (C=O), 1587 (C=C), 1262 (C-O); ^1^H-NMR (δ ppm): 1.23 (bs, 4H, -CH_2_-), 1.77 (bs, 4H, -CH_2_-), 3.77 (s, 6H, -OCH_3_), 4.33 (t, 4H, N-CH_2_), 5.23 (s, 4H, O-CH_2_), 7.33–7.35 (d, 2H, Arom. H), 7.36–7.38 (s, 2H, Arom. H), 7.52–7.54 (s, 2H, Arom. H), 8.24 (s, 2H, Arom. -1,2,3-trz. H), 9.82 (s, 2H, aldehyde-CH); ^13^C-NMR (δ ppm): 25.65 (-CH_2_-), 29.90 (-CH_2_-), 49.73 (N-CH_2_), 55.90 (-OCH_3_), 62.23 (O-CH_2_), Arom. C [110.12 (CH), 113.04 (CH), 125.33 (trz. CH), 126.26 (CH), 130.35 (C), 142.35 (C), 149.73 (trz. C-N), 153.26 (C)], 191.84 (aldehyde-CH); Anal. Calcd (%) for C_28_H_32_N_6_O_6_ (548.24): C, 61.30; H, 5.88; N, 15.32; found: C, 61.32; H, 5.89; N, 15.34; MS (ESI): *m*/*z* (%) 549.42 [M + 1]^+^.

*4-((1-(8-(4-((4-Formyl-2-methoxyphenoxy)methyl)-1H-1,2,3-triazol-1-yl)octyl)-1H-1,2,3-triazol-4-yl)-methoxy)-2-methoxybenzaldehyde* (**5e**). Yield: 78.60%; m.p. 144–145 °C; IR (ν, cm^−1^): 1690 (C=O), 1588 (C=C), 1262 (C-O); ^1^H-MR (δ ppm) : 1.05–1.19 (bs, 8H, -CH_2_-), 1.76 (m, 4H, -CH_2_-), 3.77 (s, 6H, -OCH_3_), 4.33 (t, 4H, N-CH_2_), 5.24 (s, 4H, O-CH_2_), 7.34 (s, 2H, Arom. H), 7.36–7.38 (d, 2H, Arom. H), 7.51–7.52 (d, 2H, Arom. H), 8.24 (s, 2H, Arom. -1,2,3-trz. H), 9.82 (s, 2H, aldehyde-CH); ^13^C-NMR (δ ppm): 26.17 (-CH_2_-), 28.60 (-CH_2_-), 30.08 (-CH_2_-), 49.83 (N-CH_2_), 55.87 (-OCH_3_), 62.23 (O-CH_2_), Arom. C [110.10 (CH), 113.00 (CH), 125.29 (trz. CH), 126.22 (CH), 130.36 (C), 142.38 (C), 149.73 (trz. C-N), 153.27 (C)], 191.77 (aldehyde-CH); Anal. Calcd (%) for C_30_H_36_N_6_O_6_ (576.27): C, 62.49; H, 6.29; N, 14.57; found: C, 62.48; H, 6.27; N, 14.56; MS (ESI): *m*/*z* (%) 577.36 [M + 1]^+^.

*4-((1-(10-(4-((4-Formyl-2-methoxyphenoxy)methyl)-1H-1,2,3-triazol-1-yl)decyl)-1H-1,2,3-triazol-4-yl)-methoxy)-2-methoxybenzaldehyde* (**5f**). Yield: 69.63%; m.p. 84–85 °C; IR (ν, cm^−1^): 1679 (C=O), 1586 (C=C), 1258 (C-O); ^1^H-NMR (δ ppm): 1.17 (bs, 12H, -CH_2_-), 1.77 (m, 4H, -CH_2_-), 3.78 (s, 6H, -OCH_3_), 4.33 (t, 4H, N-CH_2_), 5.23 (s, 4H, O-CH_2_), 7.35 (s, 2H, Arom. H), 7.36–7.38 (d, 2H, Arom. H), 7.52–7.54 (d, 2H, Arom. H), 8.24 (s, 2H, Arom. -1,2,3-trz. H), 9.82 (s, 2H, aldehyde-CH); ^13^C-NMR (δ ppm): 26.25 (-CH_2_-), 28.74 (-CH_2_-), 29.15 (-CH_2_-), 30.13(-CH_2_-), 49.83 (N-CH_2_), 55.91 (-OCH_3_), 62.22 (O-CH_2_), Arom. C [110.12 (CH), 113.06 (CH), 125.28 (trz. CH), 126.25 (CH), 130.35 (C), 142.30 (C), 149.74 (trz. C-N), 153.26 (C)], 191.82 (aldehyde-CH); Anal. Calcd (%) for C_32_H_40_N_6_O_6_ (604.30): C, 63.56; H, 6.67; N, 13.90; found: C, 63.5; H, 6.68; N, 13.92; MS (ESI): *m*/*z* (%) 605.36 [M + 1]^+^.

### 3.4. Biological Activity

#### 3.4.1. Acetylcholinesterase Inhibition Activity

Acetylcholinesterase inhibition activity was examined using the method described by Ellman *et al.* [20] and Inkaninan *et al.* [21], compared to galantamine as the reference drug. Firstly, all of the compounds stock solution in DMF (1 × 10^−3^ M) were prepared and then five different concentrations were prepared from stock solution in buffer (Tris-HCI pH 8.00) for experiments. Briefly 50 µL of 50 mM Tris-HCl buffer (pH 8.00), 125 µL of 3 mM DTNB (in buffer), 25 µL of 0.2 U/mL AChE and 25 µL of sample dissolved in buffer and incubated for 15 min. at 25 °C. Fifteen minutes later, 25 µL of 15 mM acetyl thiocholine iodide were added and incubated 5 min. at room temperature. The reaction was initiated by the addition of acetylcholine iodide. The hydrolysis of this substrate was monitored by the formation of the yellow 5-thio-2-nitrobenzoate anion as a result of the reaction DTNB with thiocholine, catalized by enzyme at a wave length of 412 nm using a 96-well microplate reader (Multiskan Go UV-spectrophotometer, Thermo Fisher Scientific Oy Ratastie 2, Vantaa, Finland).

Inhibition of AChE was calculated by the comparison of reaction rates of the samples relative to the blank sample (DMF in phosphate buffer pH = 8) using the Equation (1) where A_control_ is the activity of enzyme without test sample and A_sample_ is the activity of enzyme with test sample:

(1) $$\text{Scavenging effects }\left(\%\right)=\left[\frac{\left(A_{\text{control}}-A_{\text{sample}}\right)}{A_{\text{control}}}\right]\times 100$$

The experiments were carried out in triplicate and IC_50_ values were examined.

#### 3.4.2. DPPH Radical Scavenging Assay

The compounds were tested for *in vitro* antioxidant activities by the 2,2-diphenyl-1-picrylhydrazyl (DPPH) free radical scavenging assay method [22]. The total volume of the assay mixtures containing methanolic DPPH solution (0.1 mM) and different concentrations of samples was 1 mL. After incubation in the dark at room temperature for 30 min., absorbance of the samples (A_sample_) was measured at 517 nm. Assay mixture without compound was used as control (control absorbance, A_control_). All experiments were carried out in triplicate and results were expressed as the mean ±standard deviation (S.D.). Butylatedhydroxyanisole (BHA) were used as the reference compound. Free radical scavenging effect was calculated using Equation (1).

#### 3.4.3. Superoxide Radical Scavenging Assay

Superoxide scavenging activity of the compounds and reference were tested in a non-enzymatic superoxide radical (O_2_^−•^) generation assay using a modified spectrophotometrical nitro blue tetrazolium (NBT) photo reduction method [23]. The total volume of the assay mixtures containing riboflavin (2 μM), methionine (13 mM), NBT (75 mM), EDTA (0.1 mM), and the test samples (50 mM phosphate buffer, pH = 7.8) was 1 mL. After illuminating with a fluorescent lamp at 30 °C for 10 min., the absorbance of the samples (A_sample_) was measured at 560 nm. Assay mixture without the compound was used as control (control absorbance, A_control_). All experiments were carried out in triplicate and results were expressed as the mean ± standard deviation (S.D.). Free O_2_^−•^ radical scavenging effect was calculated using Equation (1).

### 3.5. Antimicrobial Activity

All test microorganisms were obtained from the Hifzissihha Institute of Refik Saydam (Ankara, Turkey) and were the following: *Escherichia coli* (*E. coli*) ATCC25922, *Yersinia pseudotuberculosis (Y. pseudotuberculosis)* ATCC911, *Pseudomonas aeruginosa* (*P. aeruginosa*) ATCC27853, *Staphylococcus aureus* *(S. aureus)* ATCC25923, *Enterococcus faecalis (E. faecalis)* ATCC29212, *Bacillus cereus (B. cereus)* 709ROMA, *Mycobacterium smegmatis (M. smegmatis)* ATCC607, *Candida albicans (C. albicans)* ATCC60193 and *Saccharomyces cerevisiae (S. cerevisiae)* RSKK251. All the extract compounds were dissolved in dimethyl sulphoxide (DMSO) to prepare chemicals stock solution of between 460–632 mg/mL.

A simple susceptibility screening test using the agar-well diffusion method [24] as adapted earlier [25] was used. Each bacterium was suspended in Mueller Hinton (MH) broth (Difco, Detroit, MI, USA). The yeast-like fungi was suspended in yeast extract broth. Then the microorganisms were diluted to approximately 10^6^ colony forming unit (cfu) per mL. For yeast-like fungi, potato dextrose agar (PDA, Difco) was used. Brain heart infusion agar (BHI, Difco, Detriot, MI, USA) was used for *M. smegmatis*. Samples were “flood-inoculated” onto the surface of MH, BHI and SD agars and then dried. Five-millimeter diameter wells were cut from the agar using a sterile cork-borer, and 50 μL of the stock extract substances were delivered into the wells. The plates were incubated for 18 h at 35 °C. The *Mycobacterium smegmatis* was grown for 3 days on BHI agar plates at 35 °C [26]. Antimicrobial activity was evaluated by measuring the zone of inhibition against the test organism. Ampicillin (10 μg), streptomycin (10 µg) and fluconazole (5 μg) were the standard drugs. Dimethyl sulphoxide was used as solvent control. The results are shown in Table 2.

## 4. Conclusions

In this study, a series of new bis-1,2,3-triazole derivatives having flexible alkyl chains connected to a phenoxy ring were synthesized and characterized. All spectra of these compounds were submitted in the supplementary materials. Antioxidant and antimicrobial assays were performed for all of the synthesized compounds. Biological activity assays (AChE inhibition, DPPH and SOD activities) were performed for all the compounds. Compound **2f** was found to display the highest AChE inhibition activity of all compounds. Compound **3b** showed a strong inhibitory effect on DPPH radicals. All other compounds showed moderate activities compared to BHA. Compound **2a** was the most effective of all compounds for SOD activity. The results of this study suggest that 1,2,3-triazole compounds have potential neuroprotective effects due to their acetylcholinesterase inhibition and antioxidant activities. All newly synthesized compounds were screened for their antibacterial activities by the inhibition zone (mm) method. All compounds were found to possess moderately antibacterial activity against the bacteria *E*. *coli*, and *Y*. *pseudotuberculosis* and antimycotic effects were observed at very low levels against *S*. *cerevisiae* and *C*. *albicans* in some molecules.

## Figures and Tables

**Table 1:** IC_50_ values of the 1,2,3-triazole compounds **2**–**5**(**a**–**f**) for AChE inhibition, DPPH and SOD activities.

Compound	AChE IC_50_ (µM)	DPPH IC_50_ (µg/mL)	SOD IC_50_ (µg/mL)
**2a**	64.65 (±2.05)	155.49 (±0.08)	45.12 (±0.04)
**2b**	52.29 (±0.16)	158.72 (±0.05)	107.54 (±0.07)
**2c**	67.03 (±1.62)	175.65 (±0.06)	147.36 (±0.05)
**2d**	62.41 (±0.85)	152.84 (±0.05)	154.79 (±0.06)
**2e**	66.38 (±2.50)	222.16 (±0.07)	131.43 (±0.06)
**2f**	50.80 (±1.01)	167.51 (±0.05)	88.15 (±0.06)
**3a**	63.28 (±0.96)	141.57 (±0.08)	120.53 (±0.04)
**3b**	56.98 (±3.87)	113.63 (±0.05)	51.37 (±0.07)
**3c**	75.70 (±6.97)	188.45 (±0.11)	284.10 (±0.07)
**3d**	75.74 (±7.19)	149.21 (±0.06)	128.24 (±0.10)
**3e**	56.99 (±0.74)	252.68 (±0.06)	52.89 (±0.06)
**3f**	78.60 (±0.81)	207.57 (±0.09)	98.35 (±0.06)
**4a**	62.82 (±1.07)	192.02 (±0.12)	55.67 (±0.08)
**4b**	74.13 (±7.5)	125.05 (±0.08)	121.74 (±0.08)
**4c**	83.37 (±1.19)	227.38 (±0.07)	60.32 (±0.07)
**4d**	58.49 (±0.72)	204.31 (±0.11)	82.77 (±0.11)
**4e**	63.39 (±0.09)	192.04 (±0.09)	88.86 (±0.09)
**4f**	62.52 (±2.55)	210.33 (±0.08)	93.47 (±0.09)
**5a**	74.69 (±0.84)	150.03 (±0.08)	126.2 (±0.07)
**5b**	61.85 (±3.54)	154.51 (±0.07)	109.17 (±0.10)
**5c**	69.41 (±0.72)	192.46 (±0.11)	145.68 (±0.11)
**5d**	70.31 (±4.65)	191.39 (±0.08)	157.14 (±0.07)
**5e**	61.81 (±2.57)	221.57 (±0.06)	188.84 (±0.08)
Galantamine	15.09 (±0.01)	-	-
BHA	-	98.56 (±0.08)	-

**Table 2:** Screening for antimicrobial activity of the compounds (μg/μL).

Compounds	Stock Concentration (µg/mL)	Microorganisms and Inhibition Zone								
		Ec	Yp	Pa	Sa	Ef	Bc	Ms	Ca	Sc
**2a**	474000	10	-	-	-	-	-	-	-	-
**2b**	488000	12	-	-	-	-	-	-	-	-
**2c**	502000	12	-	-	-	-	-	-	6	8
**2d**	516000	12	-	-	-	-	-	-	6	8
**2e**	544000	12	-	-	-	-	-	-	-	6
**2f**	572000	10	-	-	-	-	-	-	-	6
**3a**	534000	10	-	-	-	-	-	-	6	6
**3b**	548000	-	-	-	-	-	-	-	-	-
**3c**	562000	8	6	-	-	-	-	-	-	-
**3d**	576000	8	6	-	-	-	-	-	-	-
**3e**	604000	12	8	-	-	-	-	-	-	-
**3f**	632000	8	6	-	-	-	-	-	-	-
**4a**	446000	-	-	-	-	-	-	-	-	-
**4b**	460000	-	-	-	-	-	-	-	6	6
**4c**	474000	10	-	-	-	-	-	-	6	6
**4d**	488000	-	-	-	-	-	-	-	-	6
**4e**	516000	-	-	-	-	-	-	-	6	6
**4f**	544000	8	8	-	-	-	-	-	-	-
**5a**	506000	12	8	-	-	-	-	-	-	-
**5b**	520000	10	8	-	-	-	-	-	-	-
**5c**	534000	10	8	-	-	-	-	-	-	-
**5d**	548000	10	8	-	-	-	-	-	-	-
**5e**	576000	10	6	-	-	-	-	-	-	-
**5f**	604000	8	6	-	-	-	-	-	-	-
	Amp	10	10	18	10	35	15			
	Strep							35		
	Flu.								25	25

Ec: *E. coli* ATCC 35218; Yp: *Y. pseudotuberculosis* ATCC 911; Pa: *P. aeruginosa* ATCC 10145; Sa: *S. aureus* ATCC 25923; Ef: *E. faecalis* ATCC 29212; Bc: *B. cereus* 709 Roma; Ms: *M. smegmatis* ATCC607; Ca: *C. albicans* ATCC 60193; *S. cerevisiae* RSKK 251; Amp.: Ampicillin; Strep.: Streptomycin; Flu.: Fluconazole; (-): no activity of test concentrations.

## Supplementary Materials

Supplementary materials can be accessed at: http://www.mdpi.com/1420-3049/21/5/659/s1.
