# Supplementary material for: Synthesis of Novel Symmetrical 1,4-Disubstituted 1,2,3-Bistriazole Derivatives via ‘Click Chemistry’ and Their Biological Evaluation
Source: Molecules. 2016 May 19;21(5):659. doi: 10.3390/molecules21050659 (PMC6274262; doi:10.3390/molecules21050659)
Supplement: Supplementary file 1 [file molecules-21-00659-s001.pdf]

SupplementaryMaterials: Synthesis of Novel Symmetrical 1,4-Disubstited 1,2,3-Bistriazole Derivatives via 'Click Chemistry' and Biological Evaluation

EsraDüğdü,DilekÜnlüer,FatihÇelik,KemalSancak,ŞengülAlpayKaraoğluandArzuÖzel

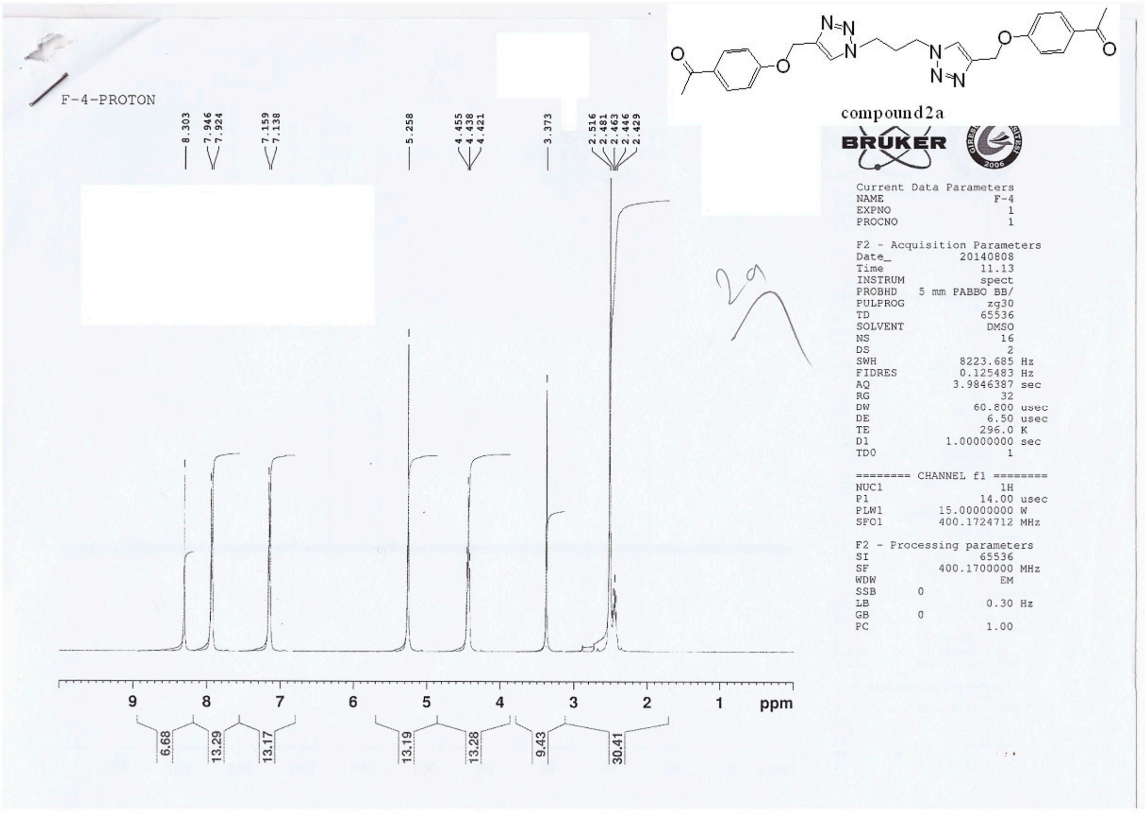

Figure S1. ^{1}H-NMR spectrum of compound **2a**.

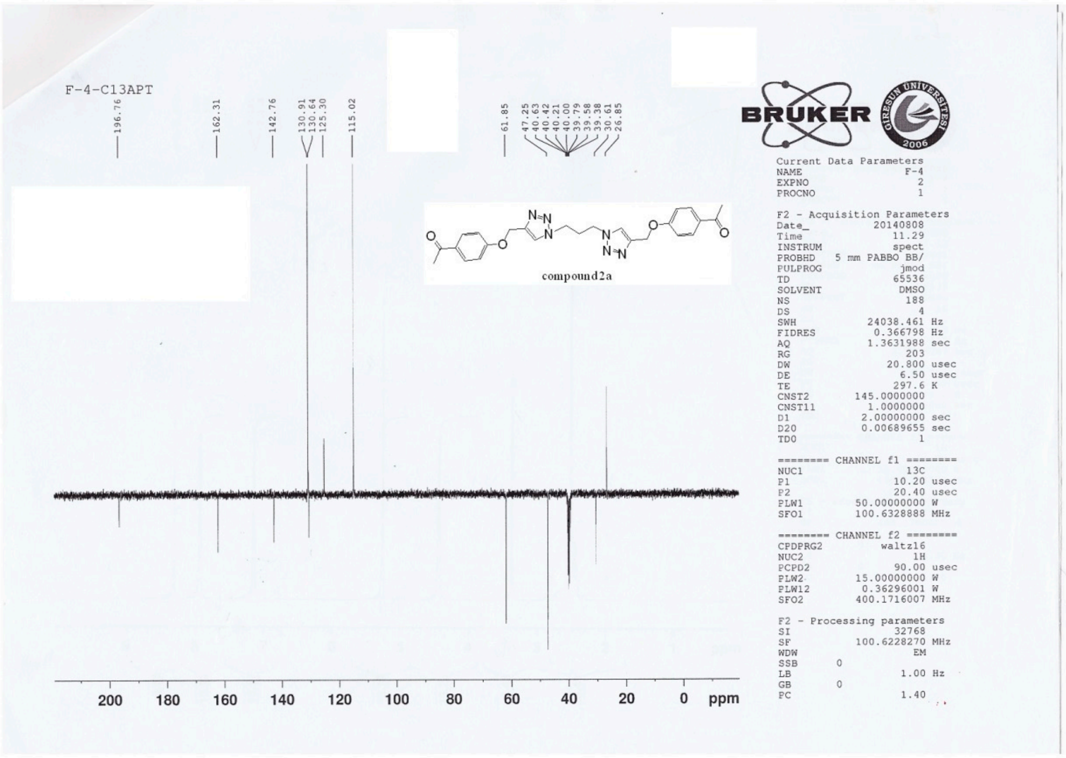

Figure S2. ^{13}C-NMR spectrumofcompound **2a**.

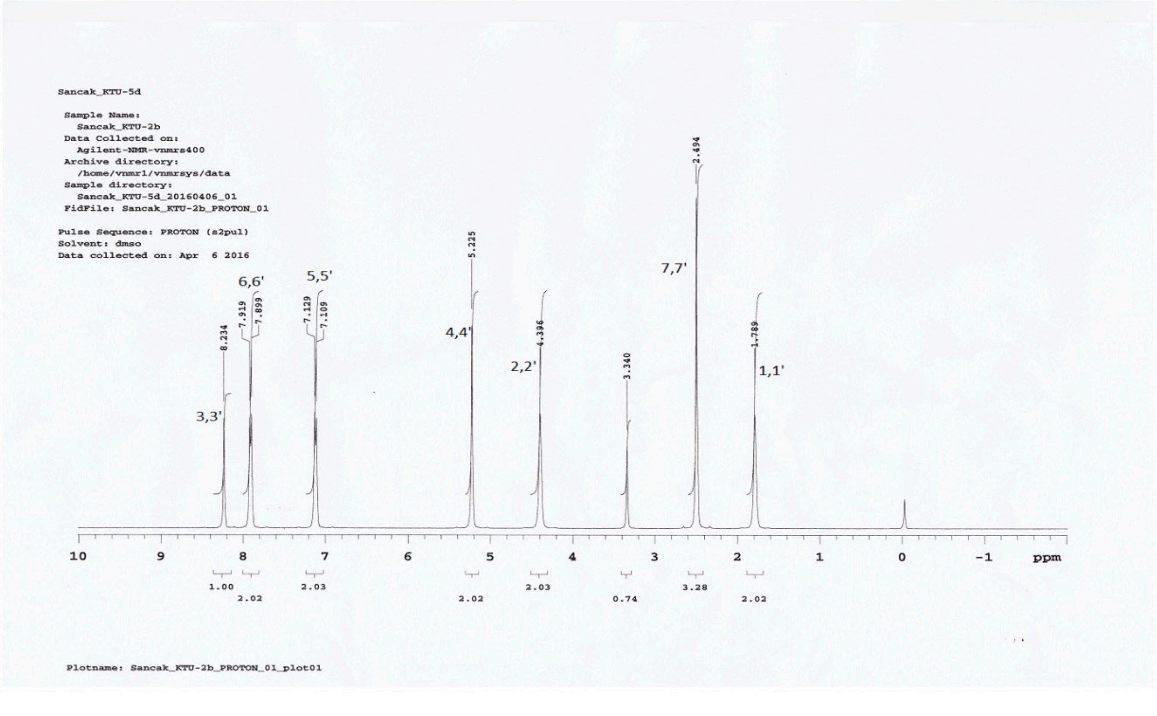

Figure S3. ^{1}H-NMR spectrum of compound **2b**.

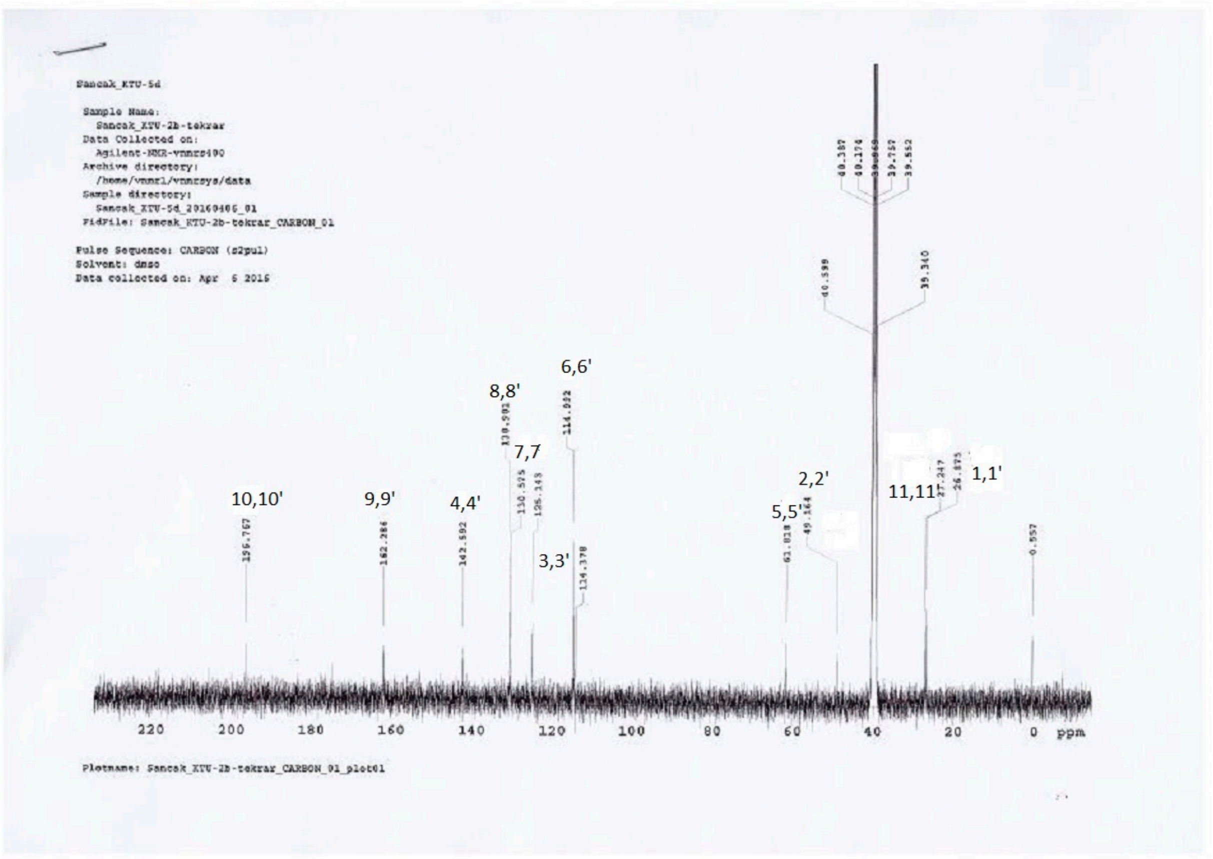

Figure S4. ^{13}C-NMR spectrum of compound **2b**.

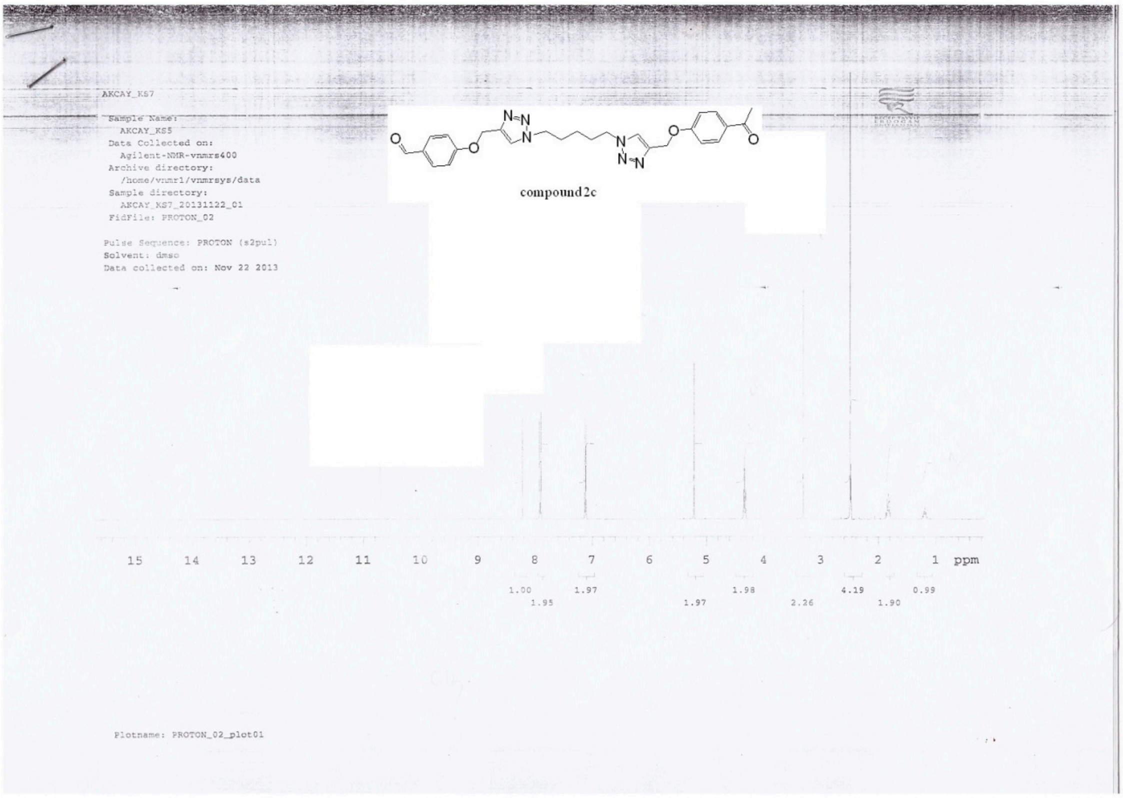

Figure S5. ^{1}H-NMR spectrum of compound **2c**.

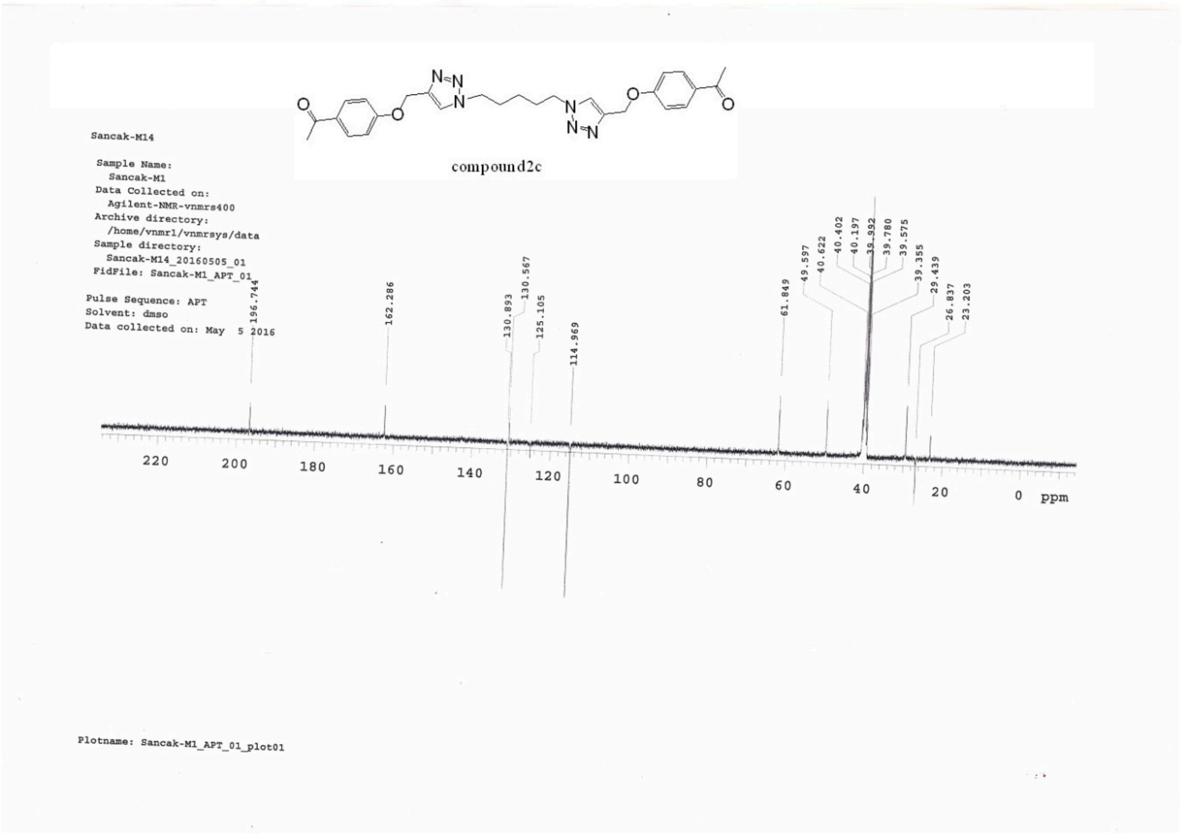

Figure S6. ^{13}C-NMR spectrum of compound **2c**.

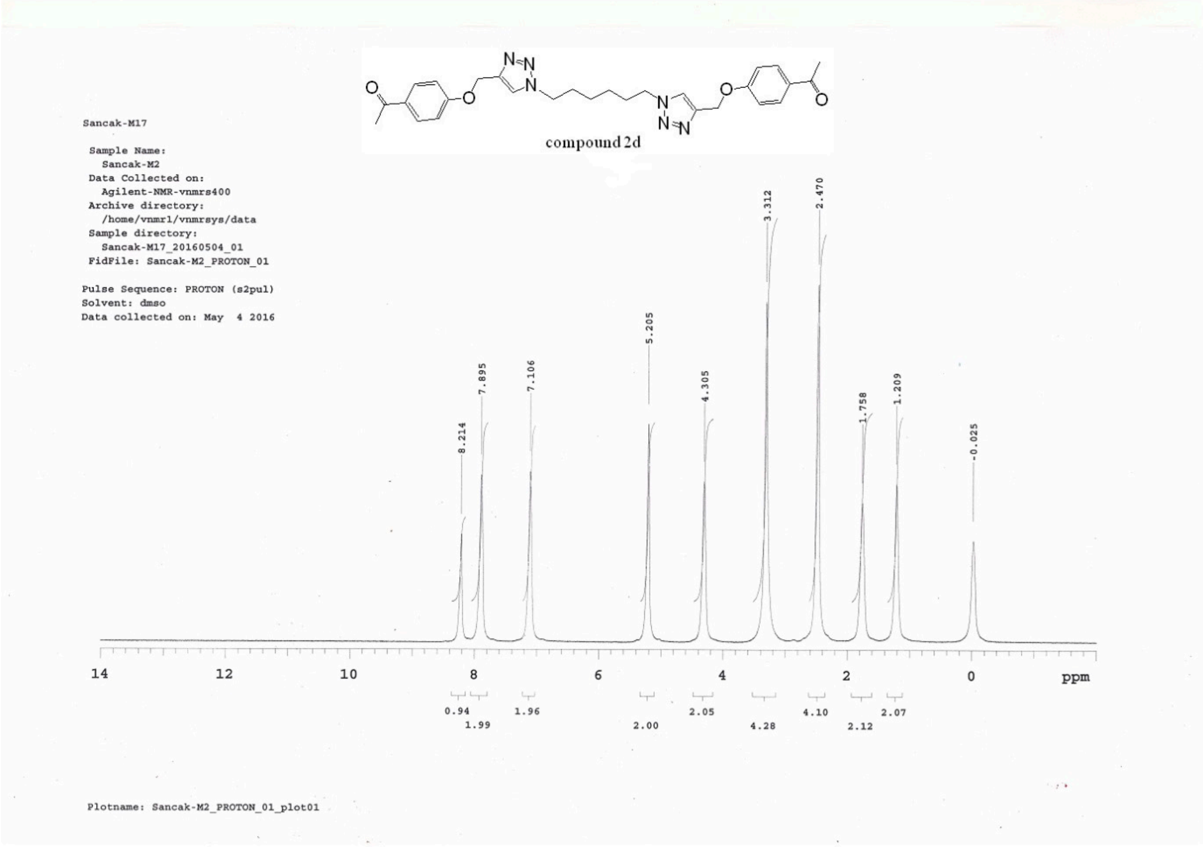

Figure S7. ^{1}H-NMR spectrum of compound **2d**.

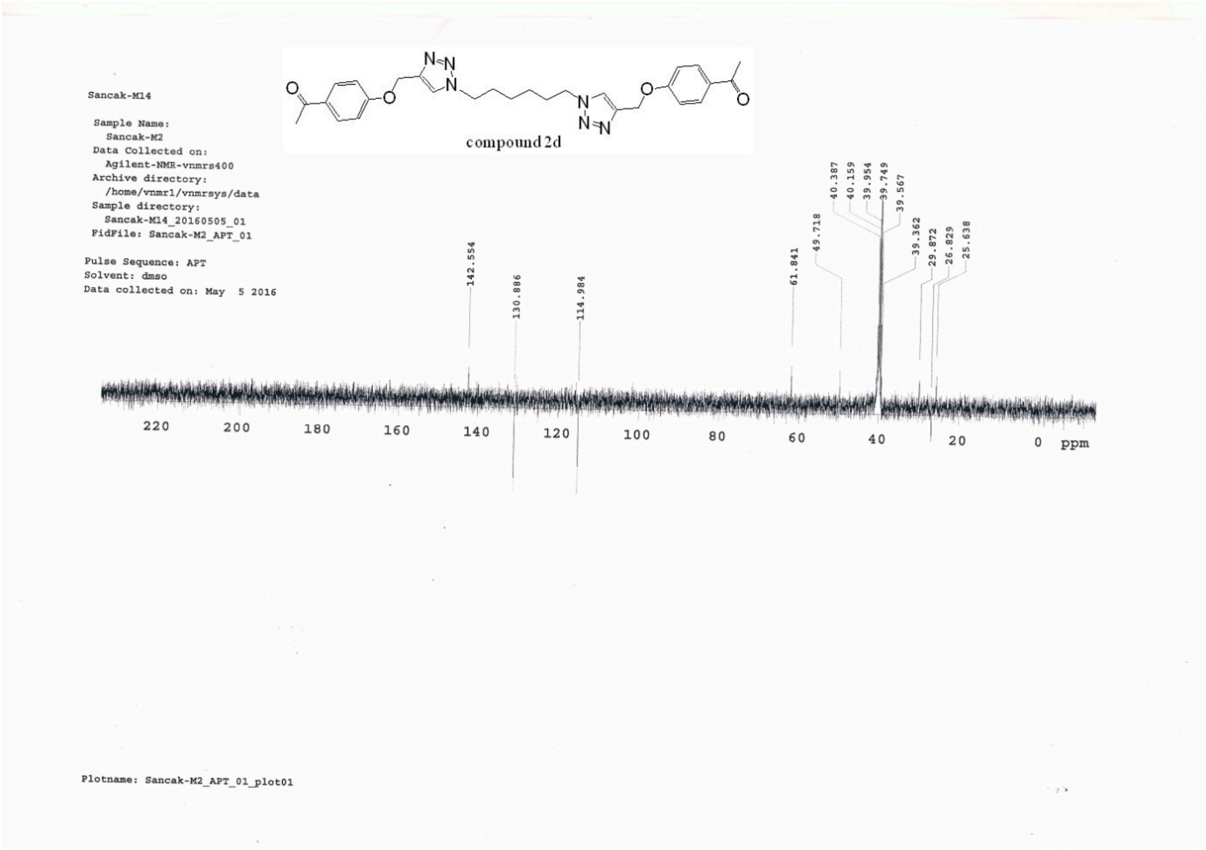

Figure S8. ^{13}C-NMR spectrum of compound **2d**.

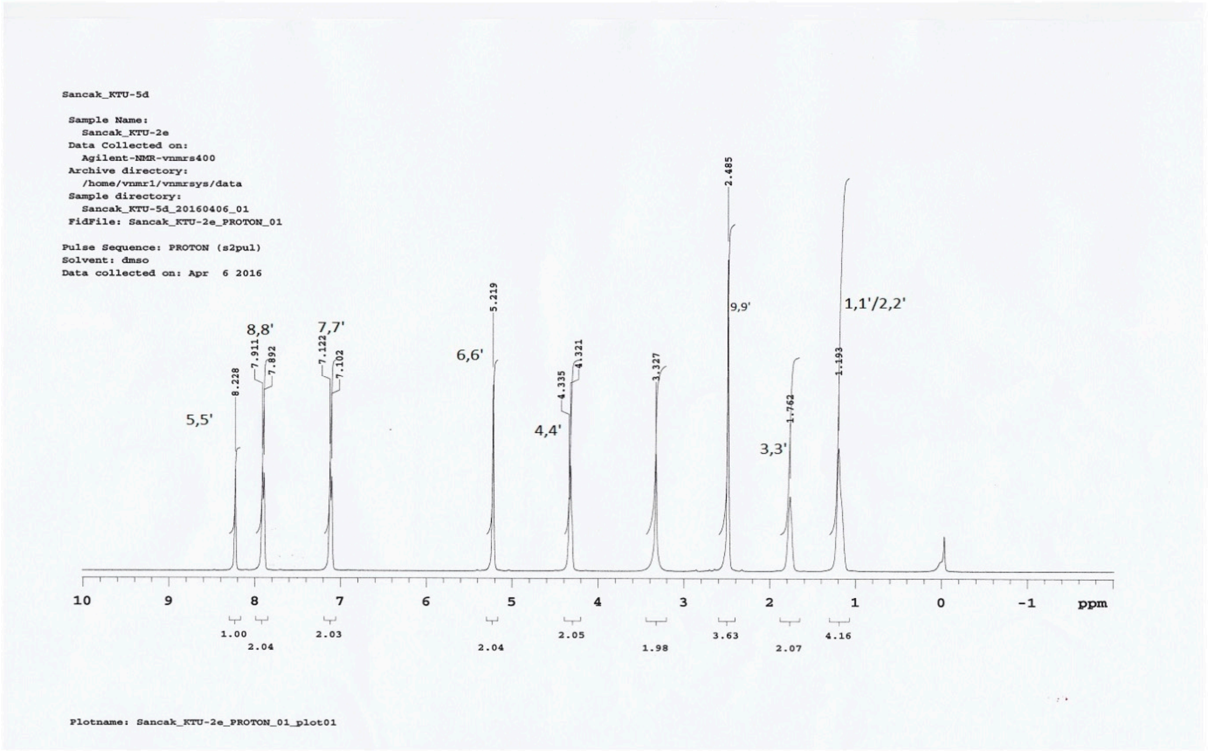

Figure S9. ^{1}H-NMR spectrum of compound **2e**.

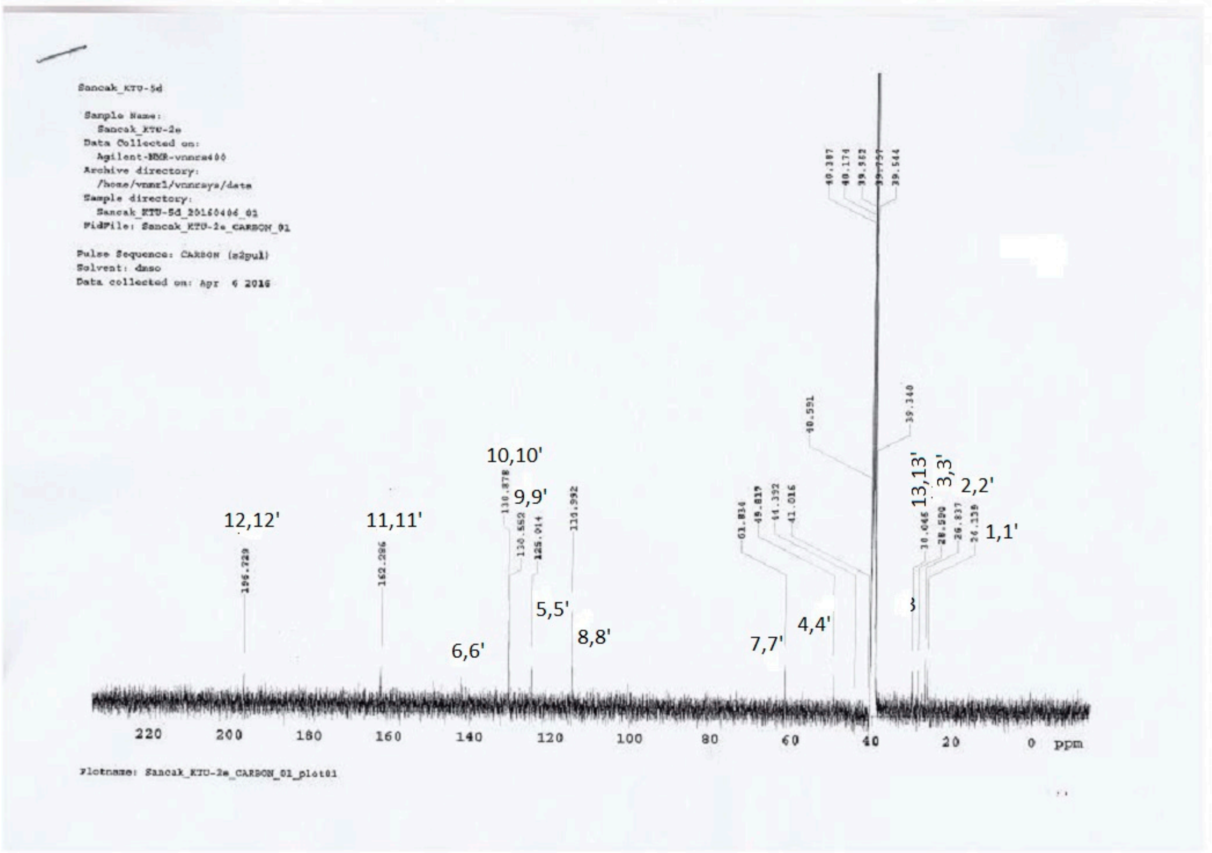

Figure S10. ^{13}C-NMR spectrum of compound **2e**.

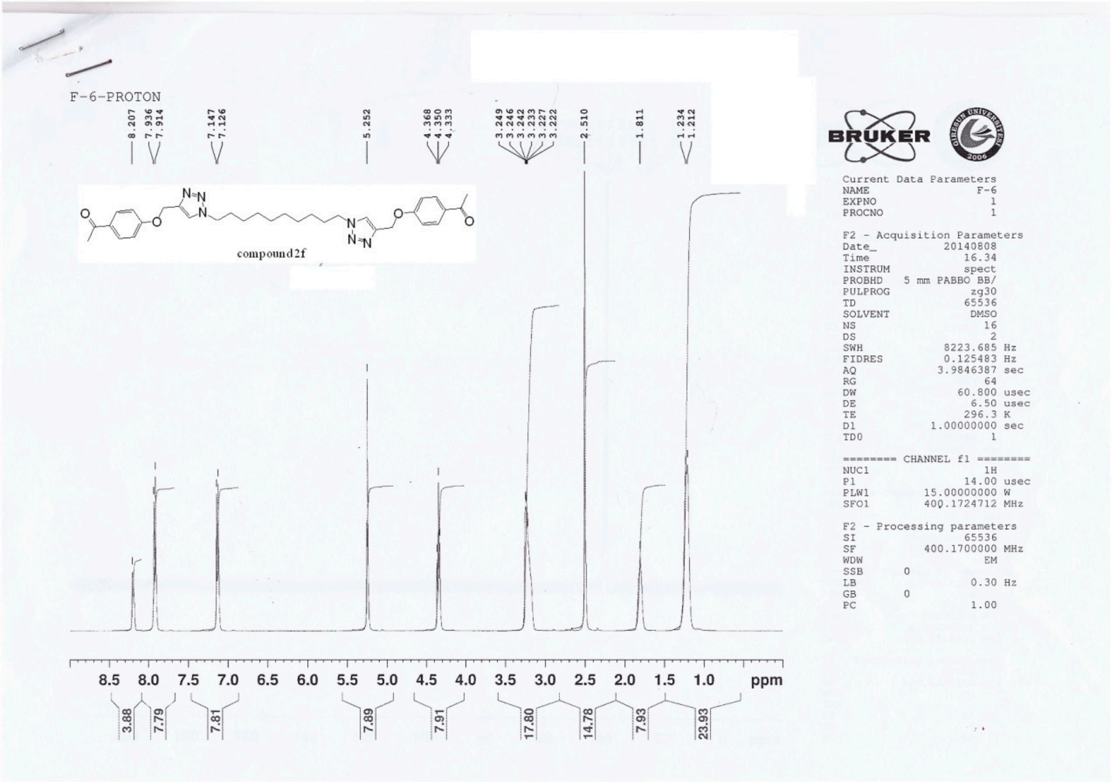

Figure S11. ^{1}H-NMR spectrum of compound **2f**.

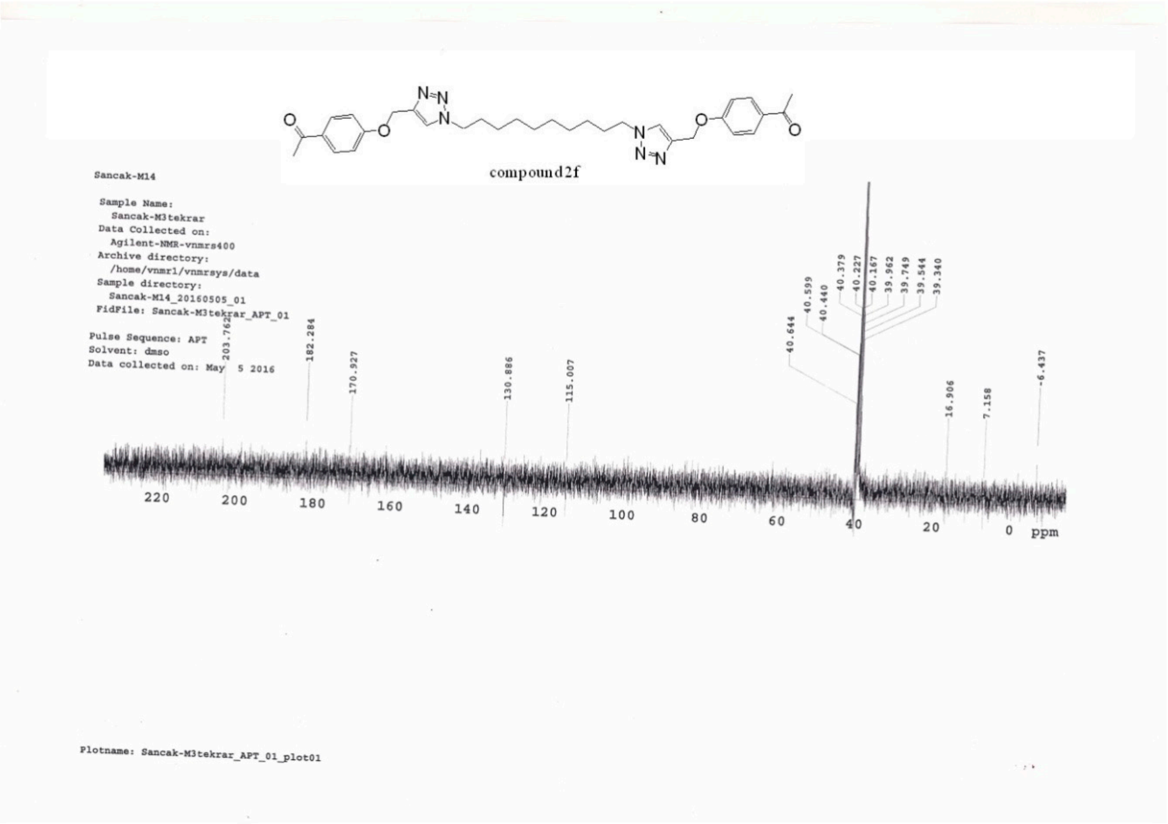

Figure S12. ^{13}C-NMR spectrum of compound **2f**.

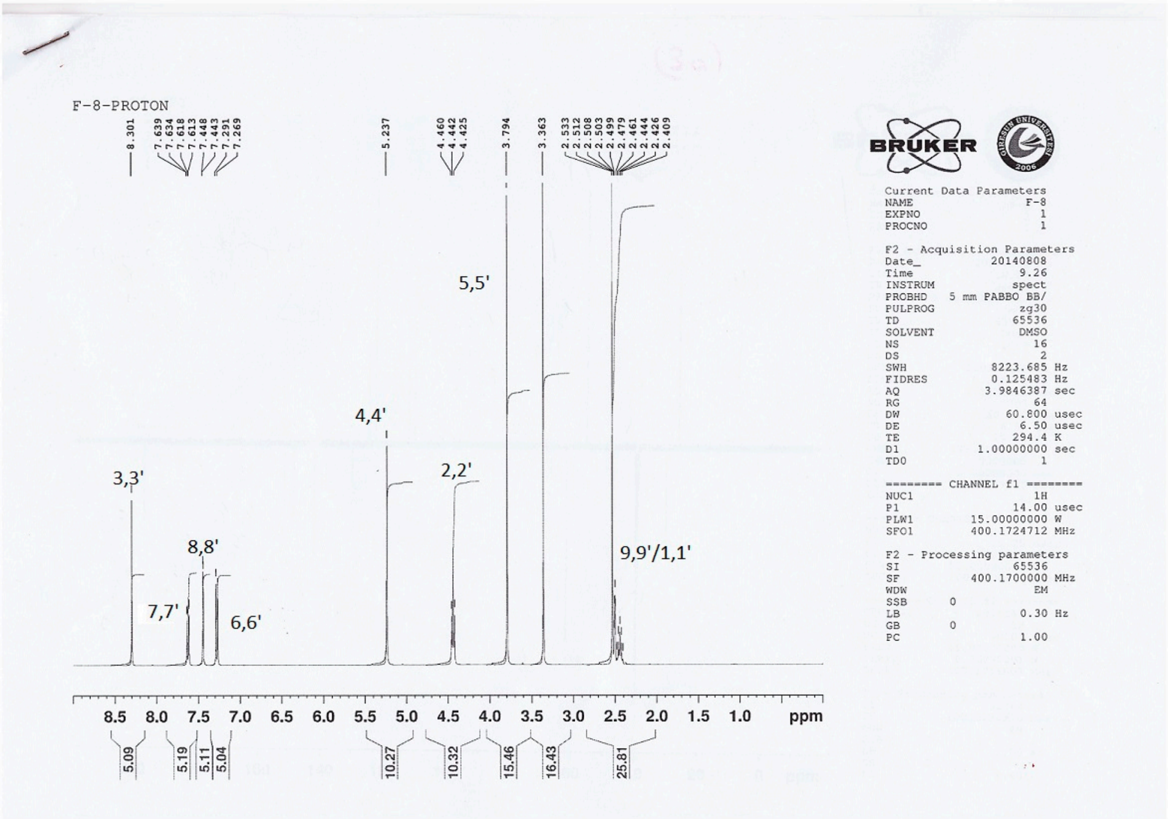

Figure S13. ^{1}H-NMR spectrum of compound **3a**.

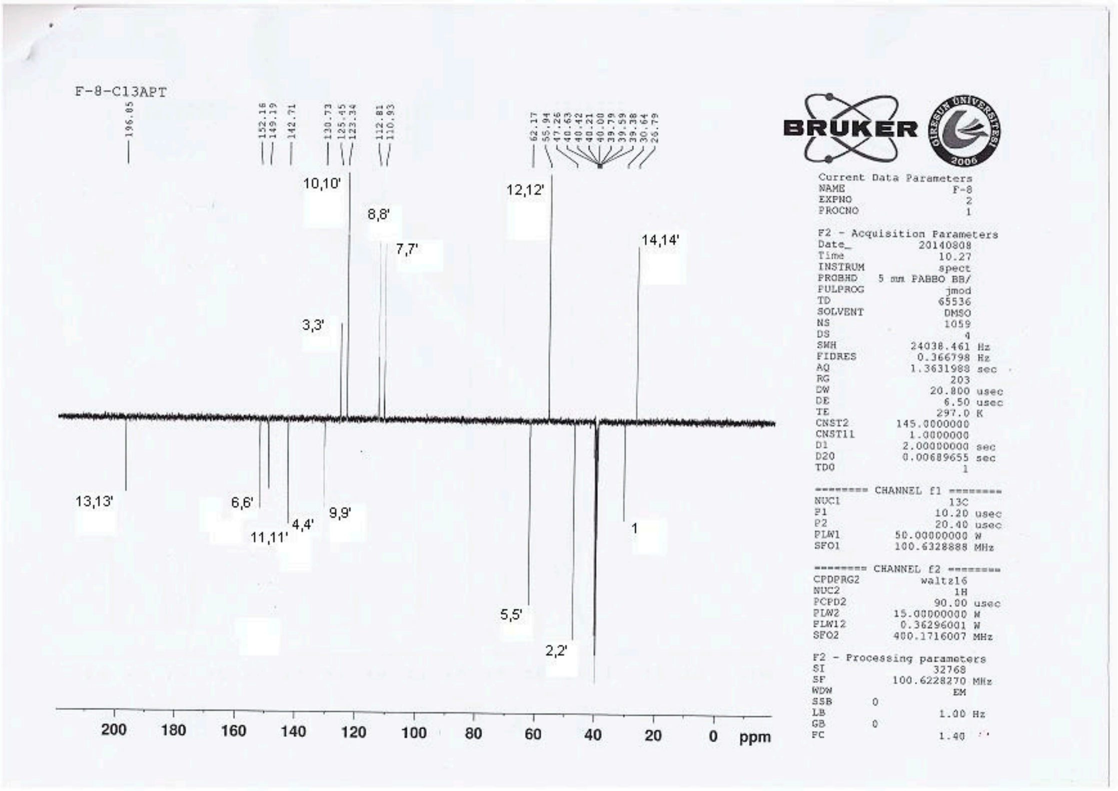

Figure S14. ^{13}C-NMR spectrum of compound **3a**.

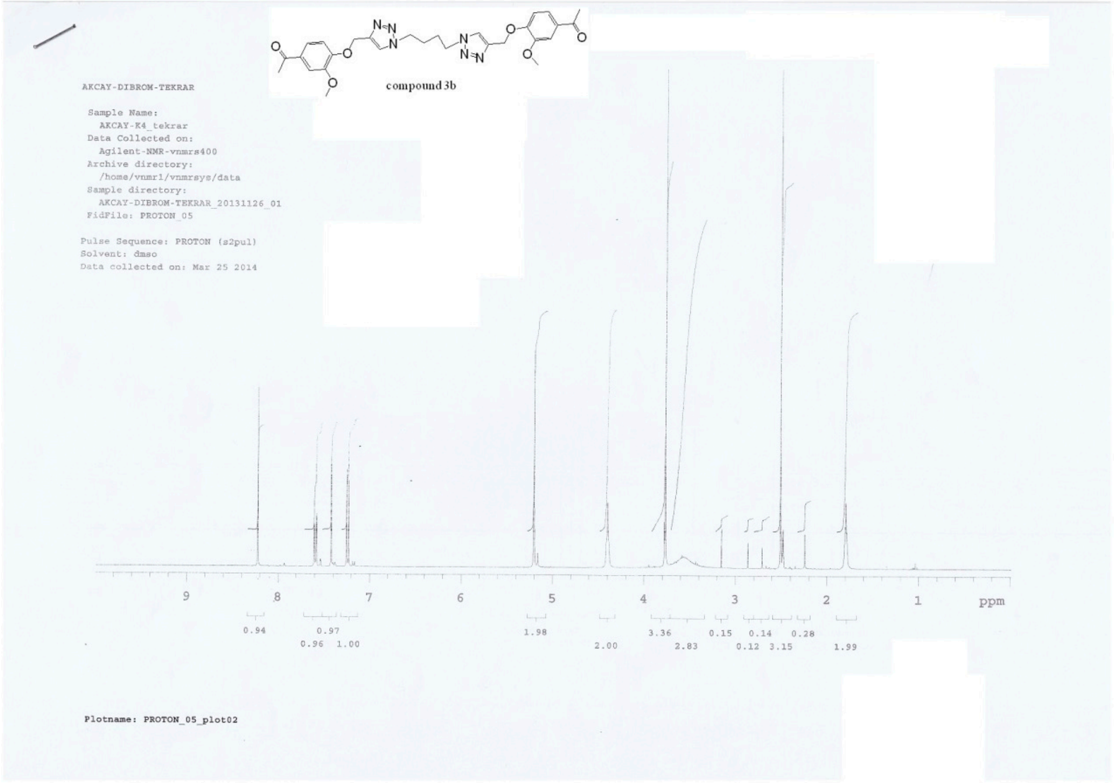

Figure S15. ^{1}H-NMR spectrum of compound **3b**.

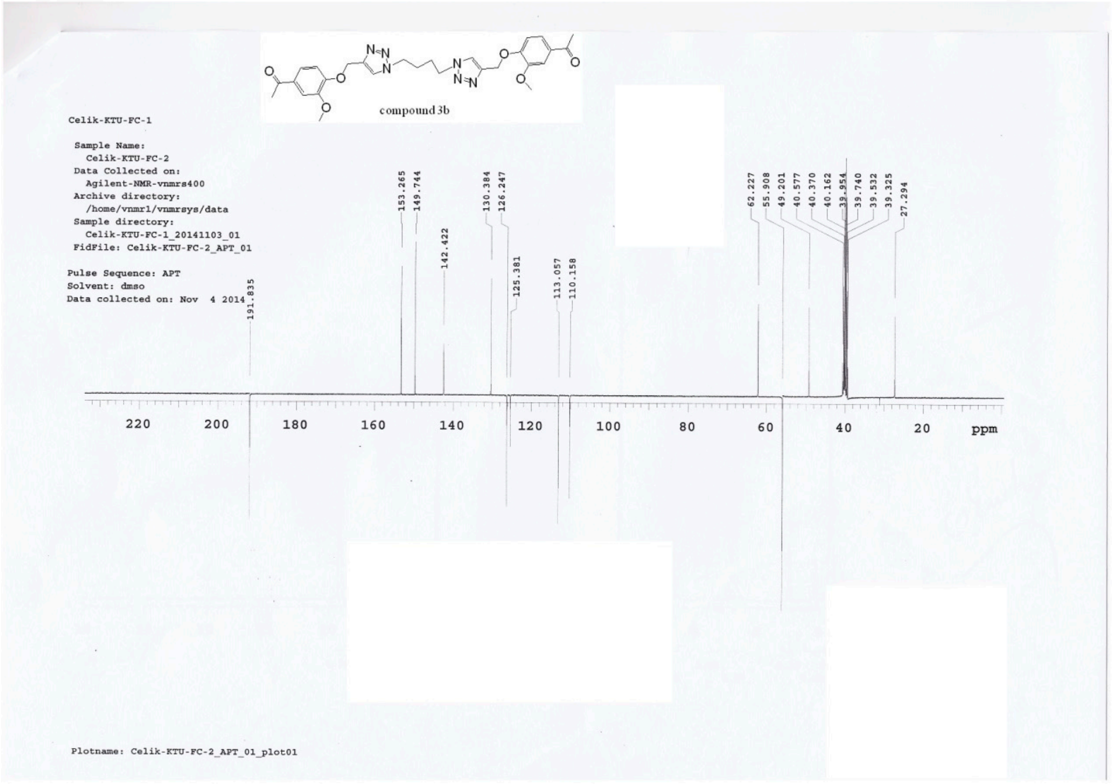

Figure S16. ^{13}C-NMR spectrum of compound **3b**.

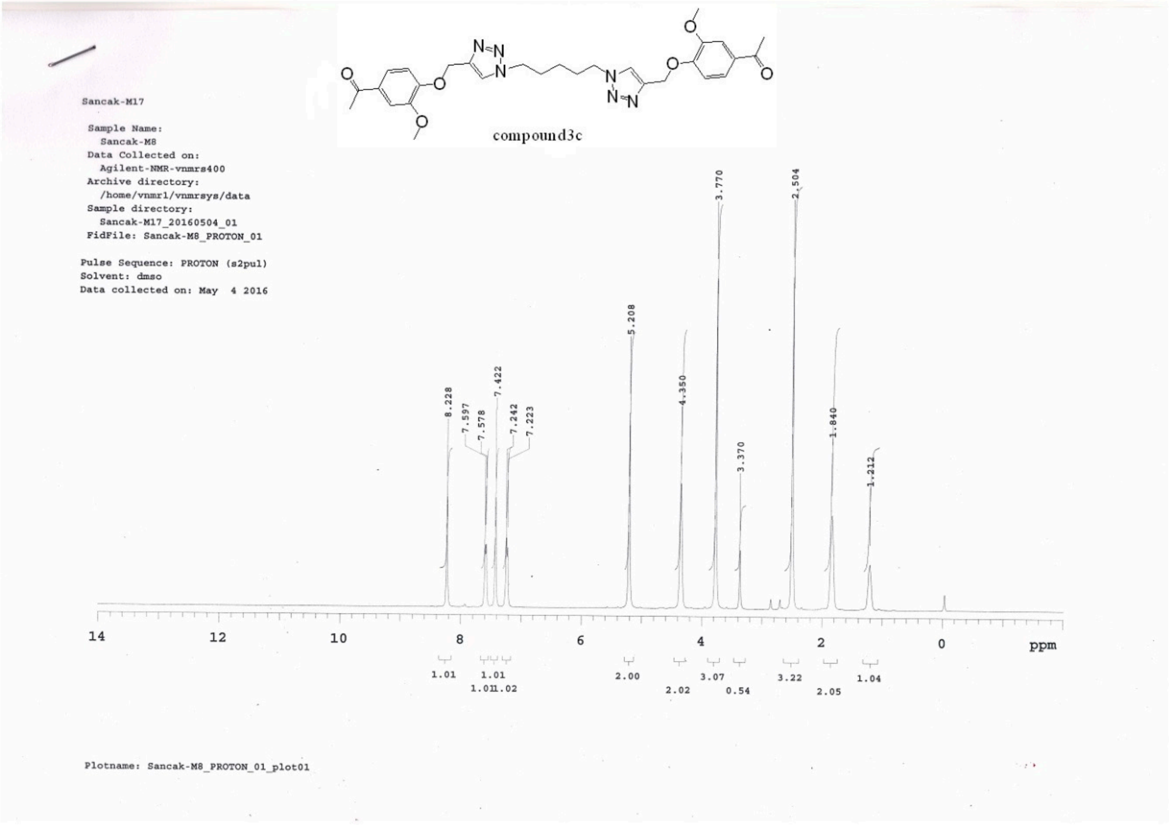

Figure S17. ^{1}H-NMR spectrum of compound **3c**.

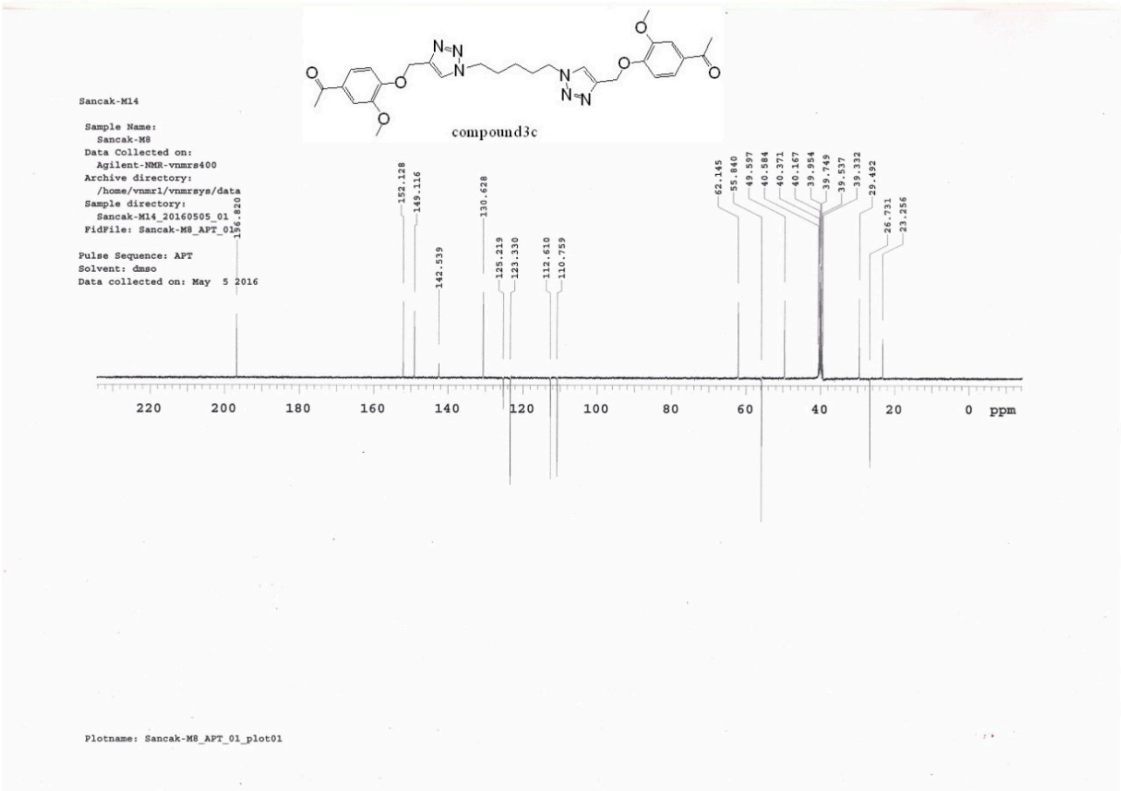

Figure S18. ^{13}C-NMR spectrum of compound **3c**.

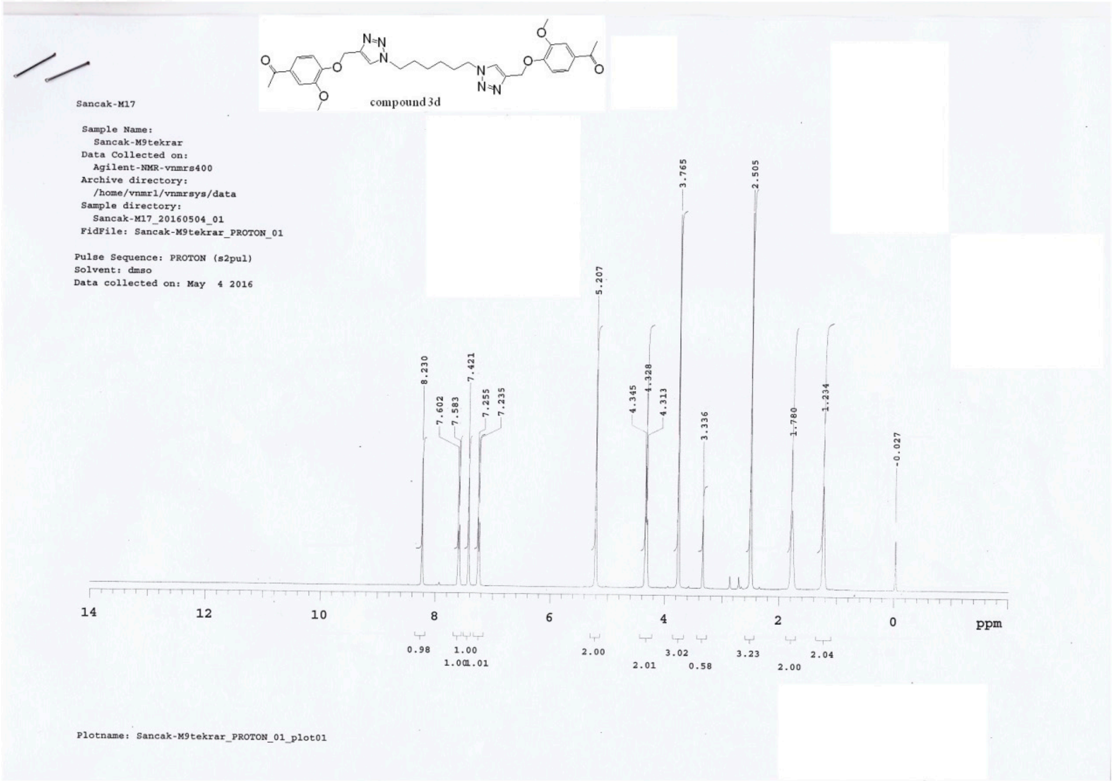

Figure S19. ^{1}H-NMR spectrum of compound **3d**.

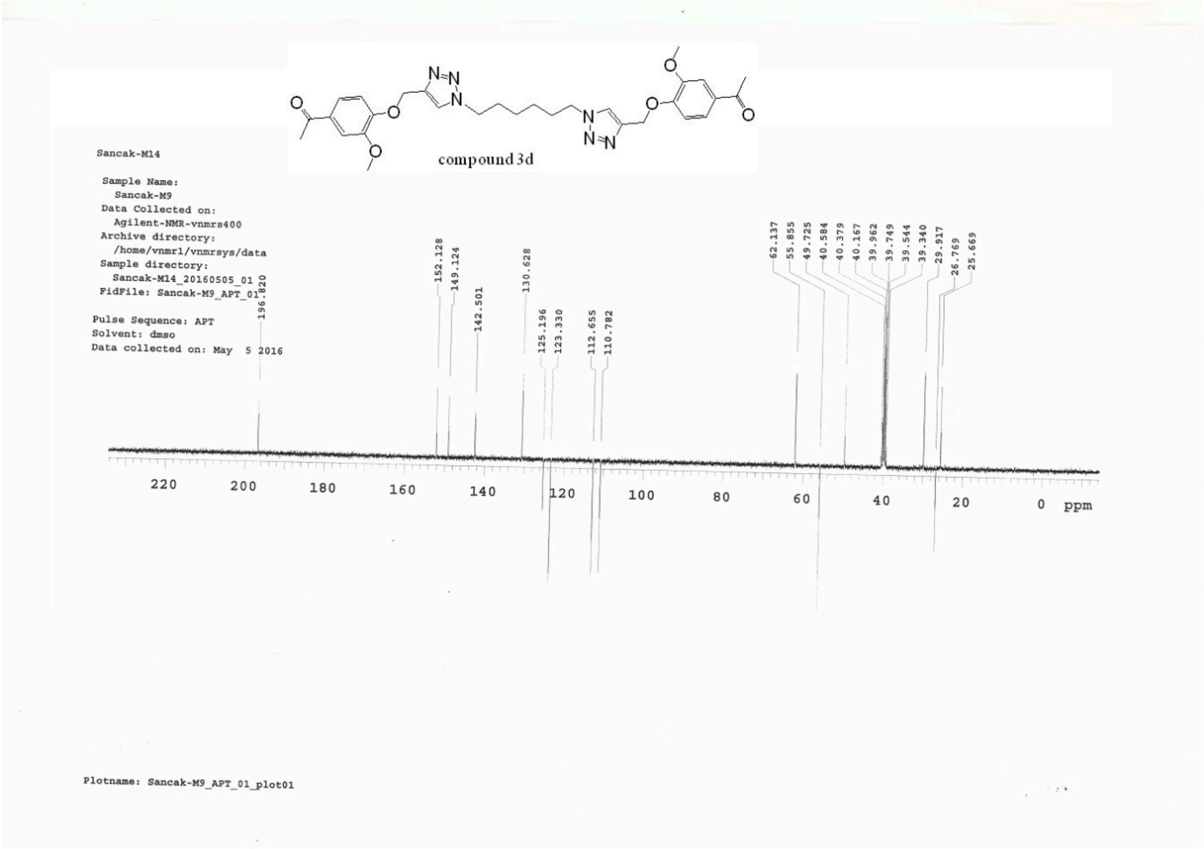

Figure S20. ^{13}C-NMR spectrum of compound **3d**.

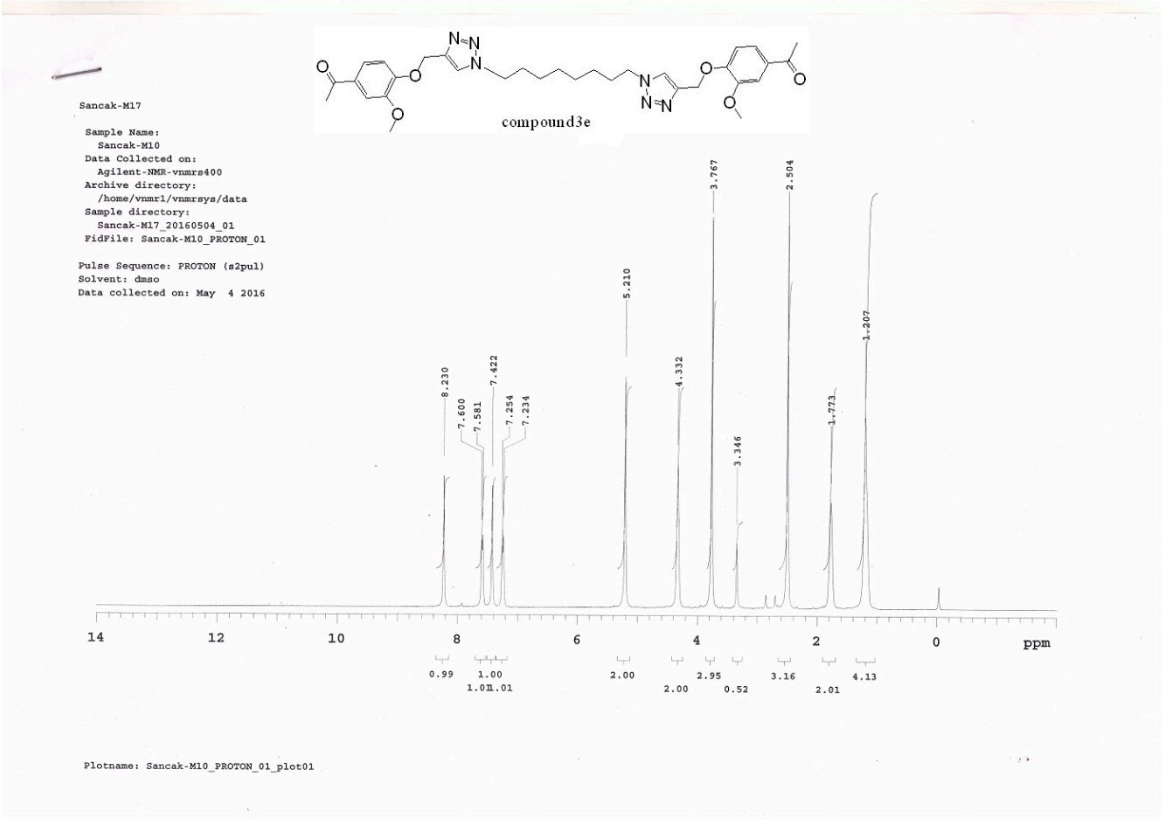

Figure S21. ^{1}H-NMR spectrum of compound **3e**.

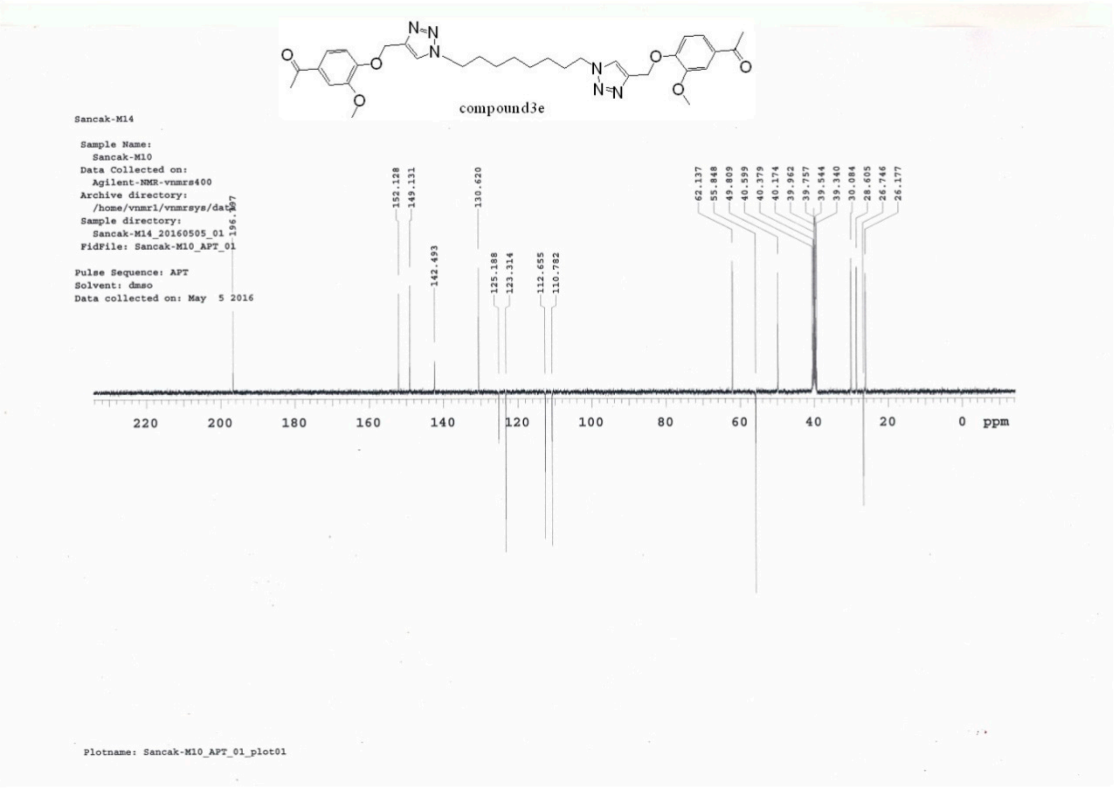

Figure S22. ^{13}C-NMR spectrum of compound **3e**.

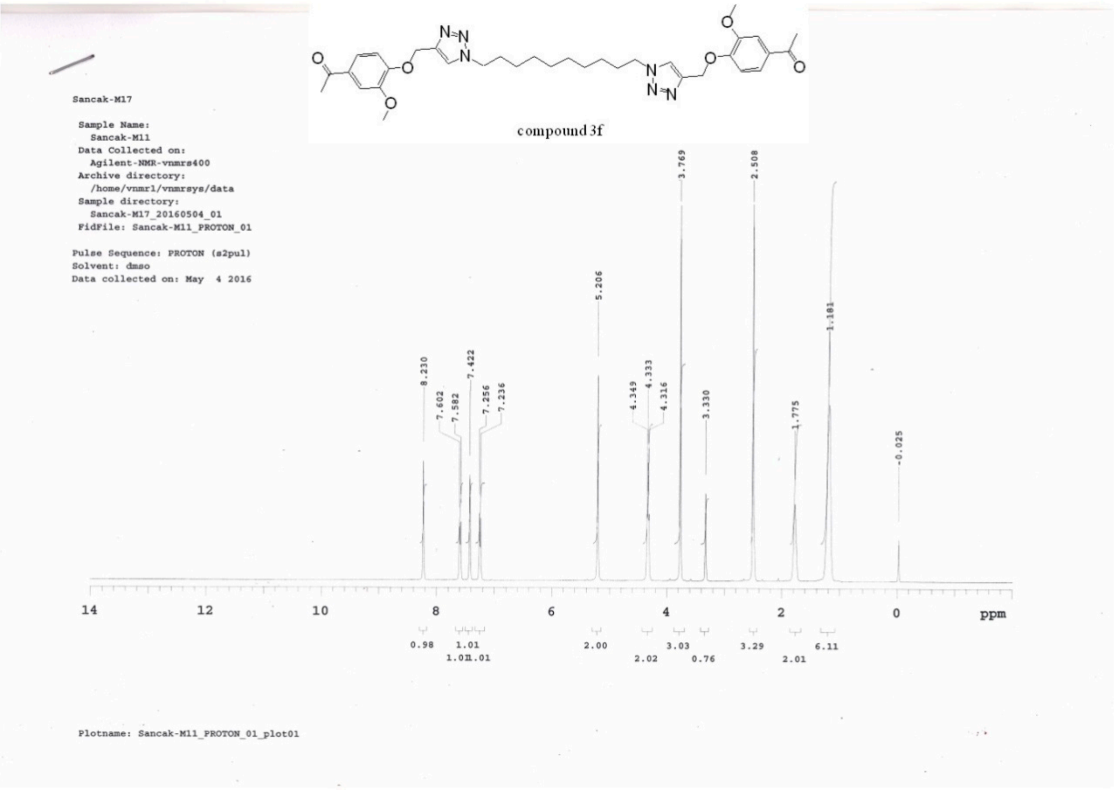

Figure S23. ^{1}H-NMR spectrum of compound **3f**.

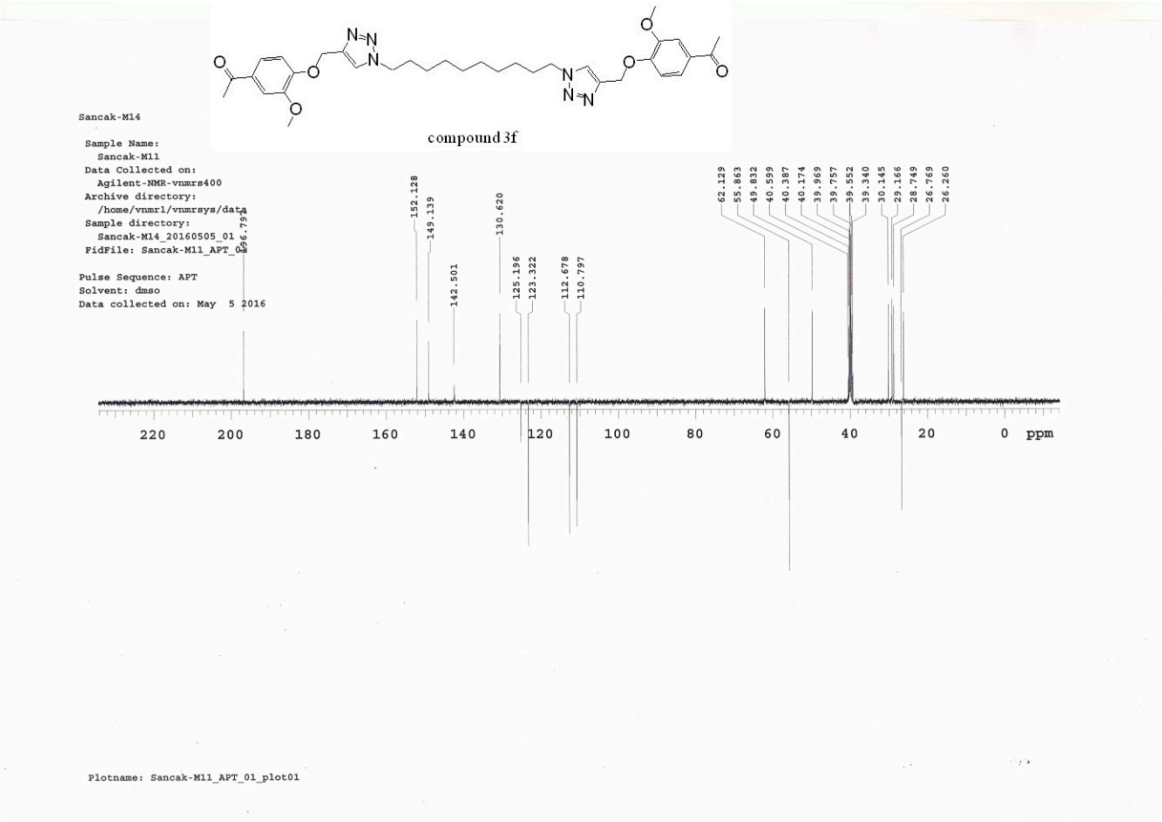

Figure S24. ^{13}C-NMR spectrum of compound **3f**.

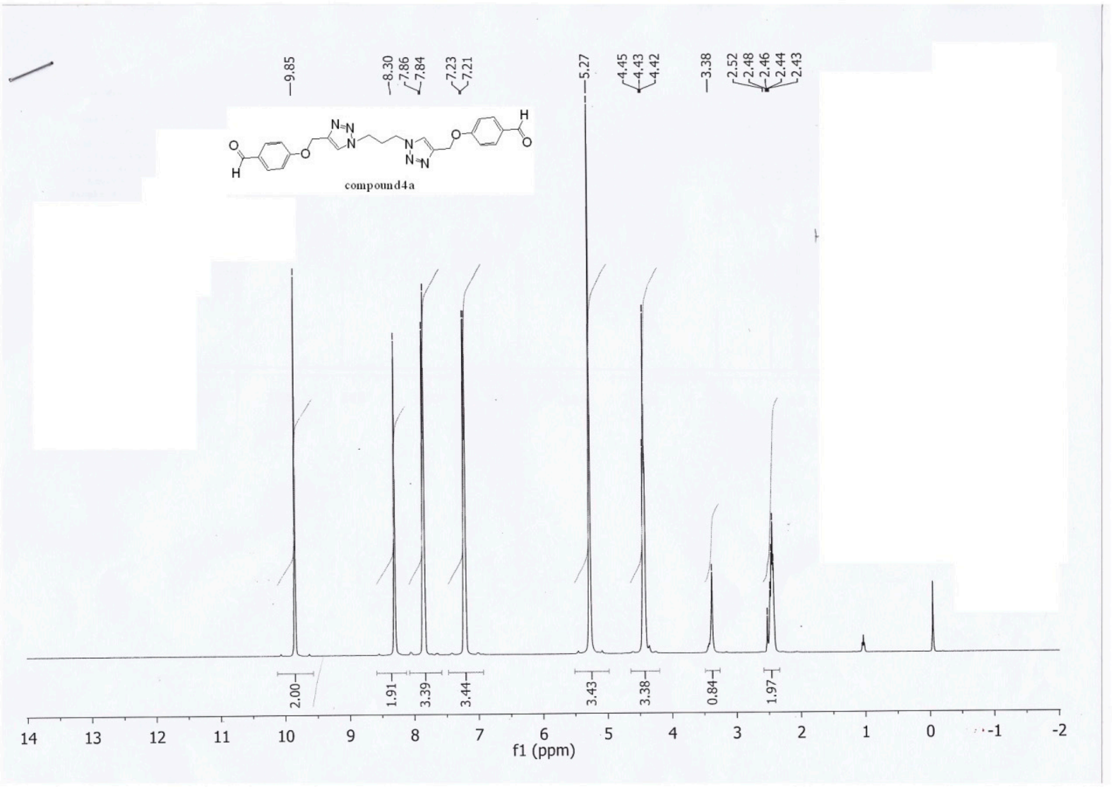

Figure S25. ^{1}H-NMR spectrum of compound **4a**.

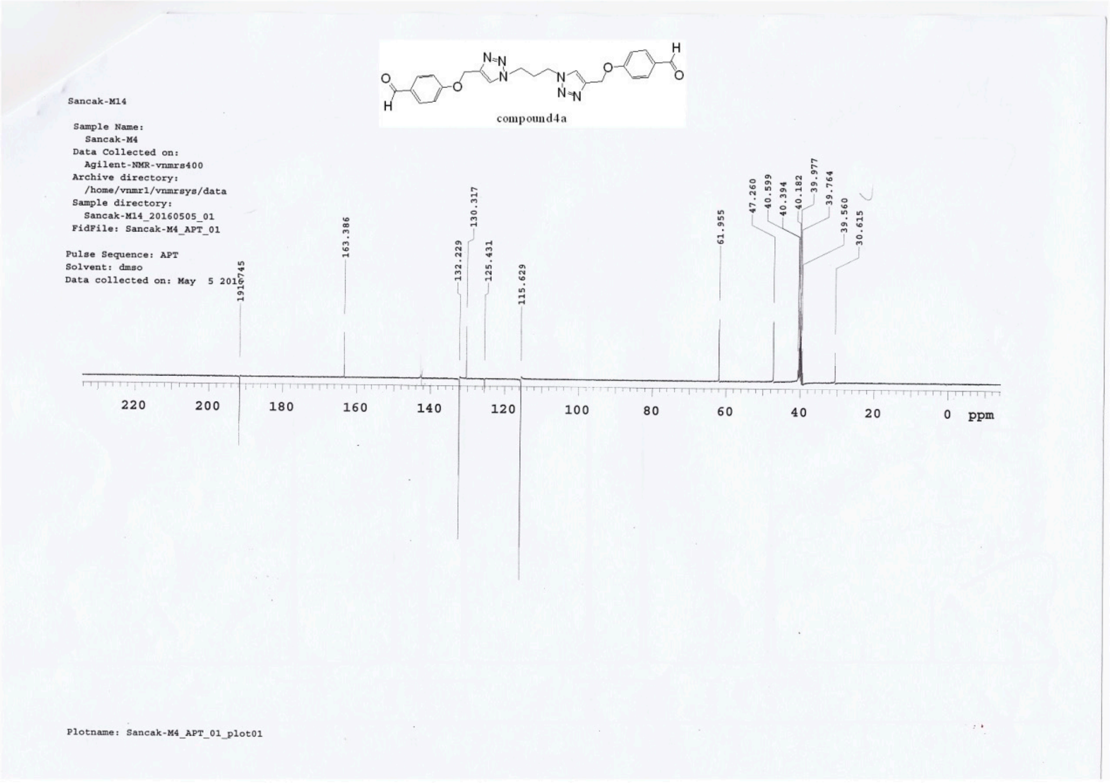

Figure S26. ^{13}C-NMR spectrum of compound **4a**.

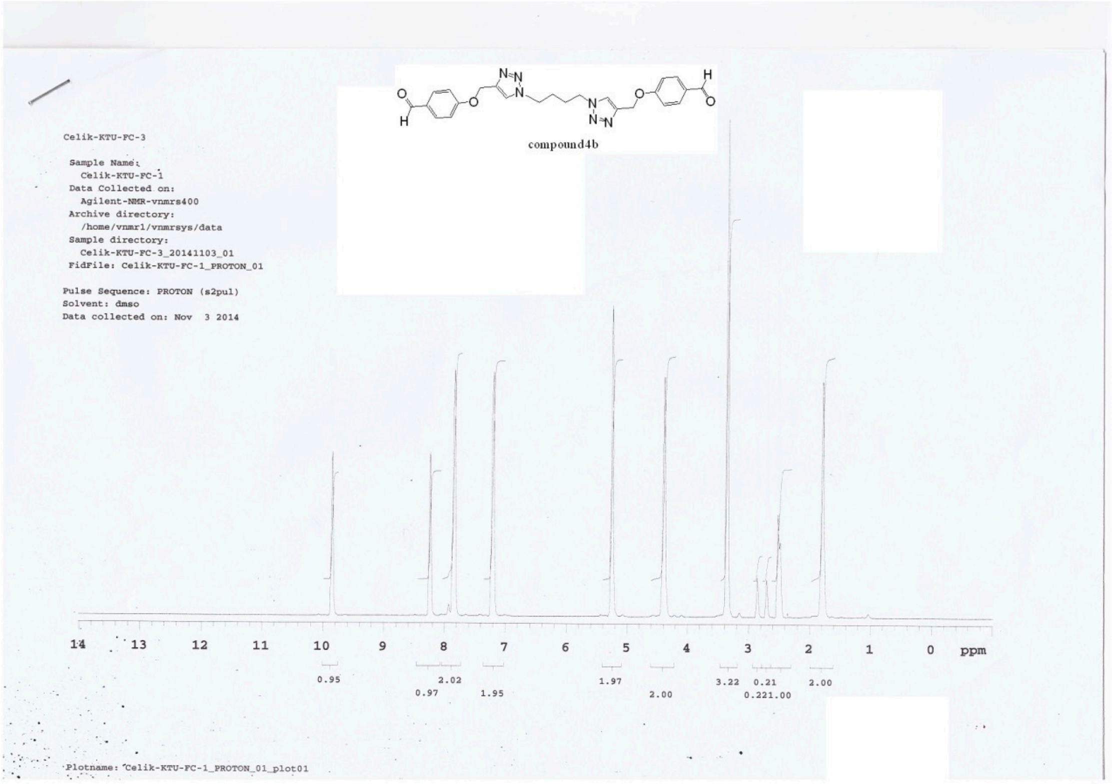

Figure S27. ^{1}H-NMR spectrum of compound **4b**.

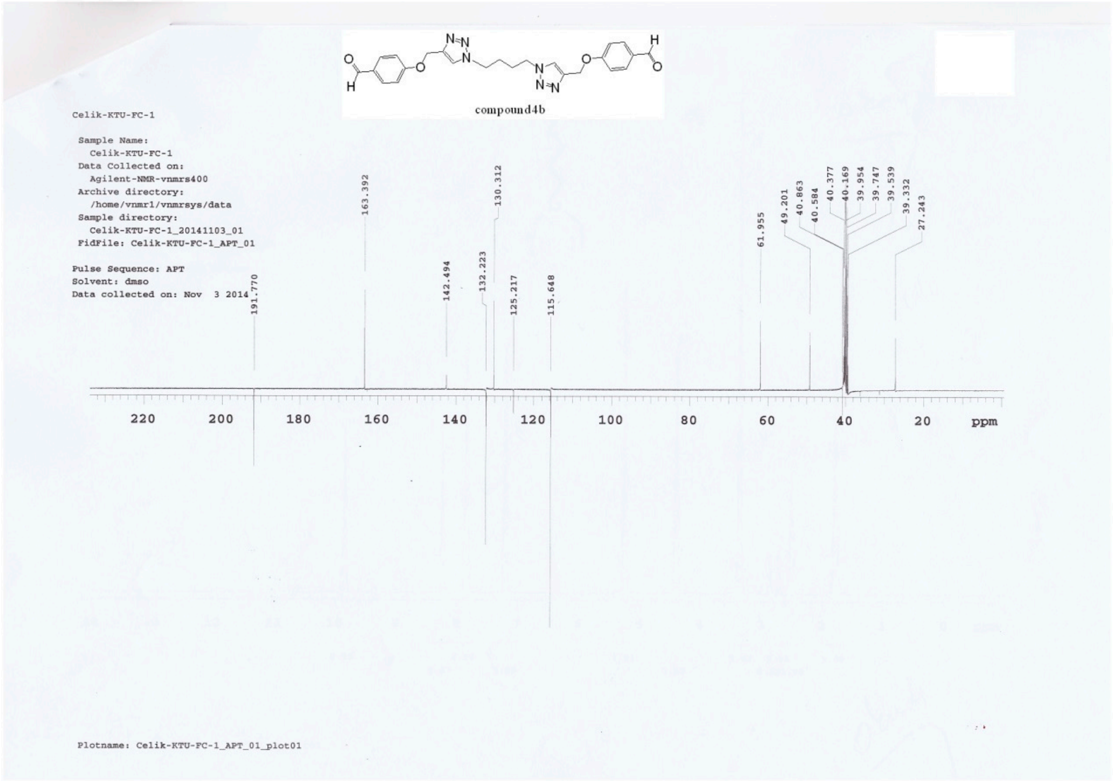

Figure S28. ^{13}C-NMR spectrum of compound **4b**.

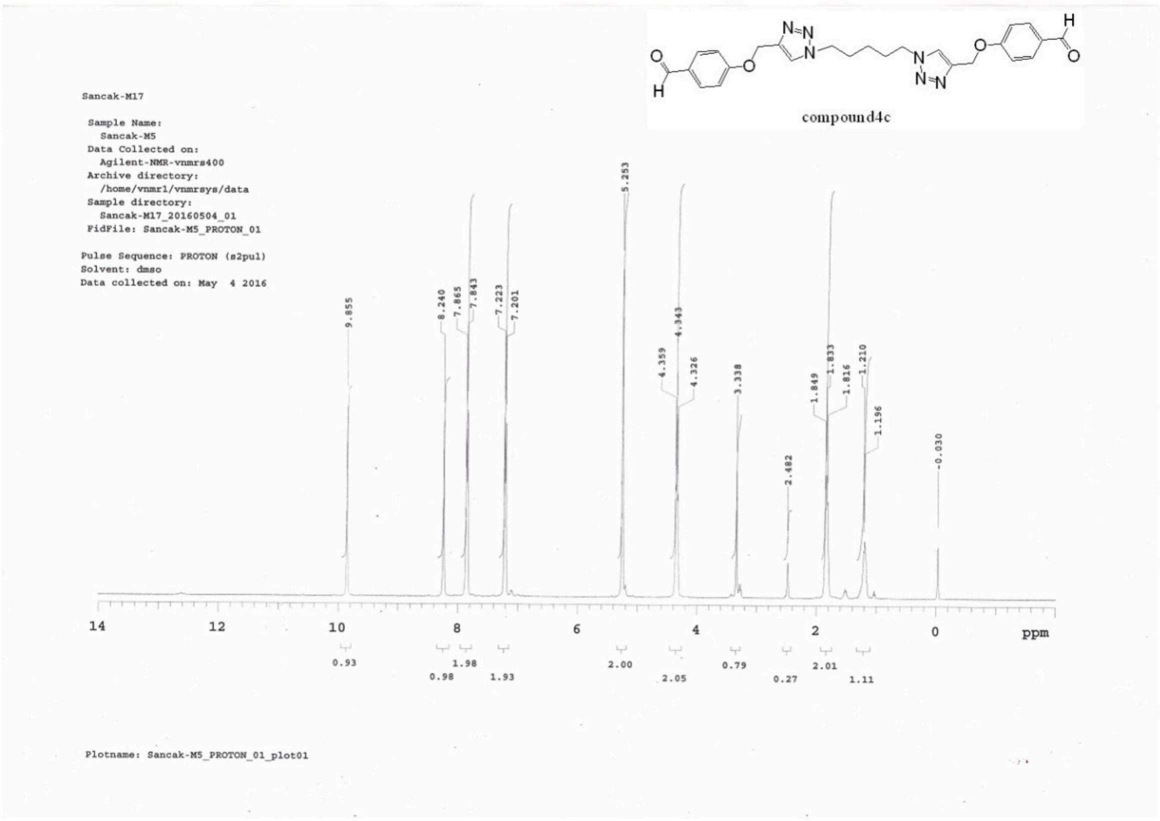

Figure S29. ^{1}H-NMR spectrum of compound **4c**.

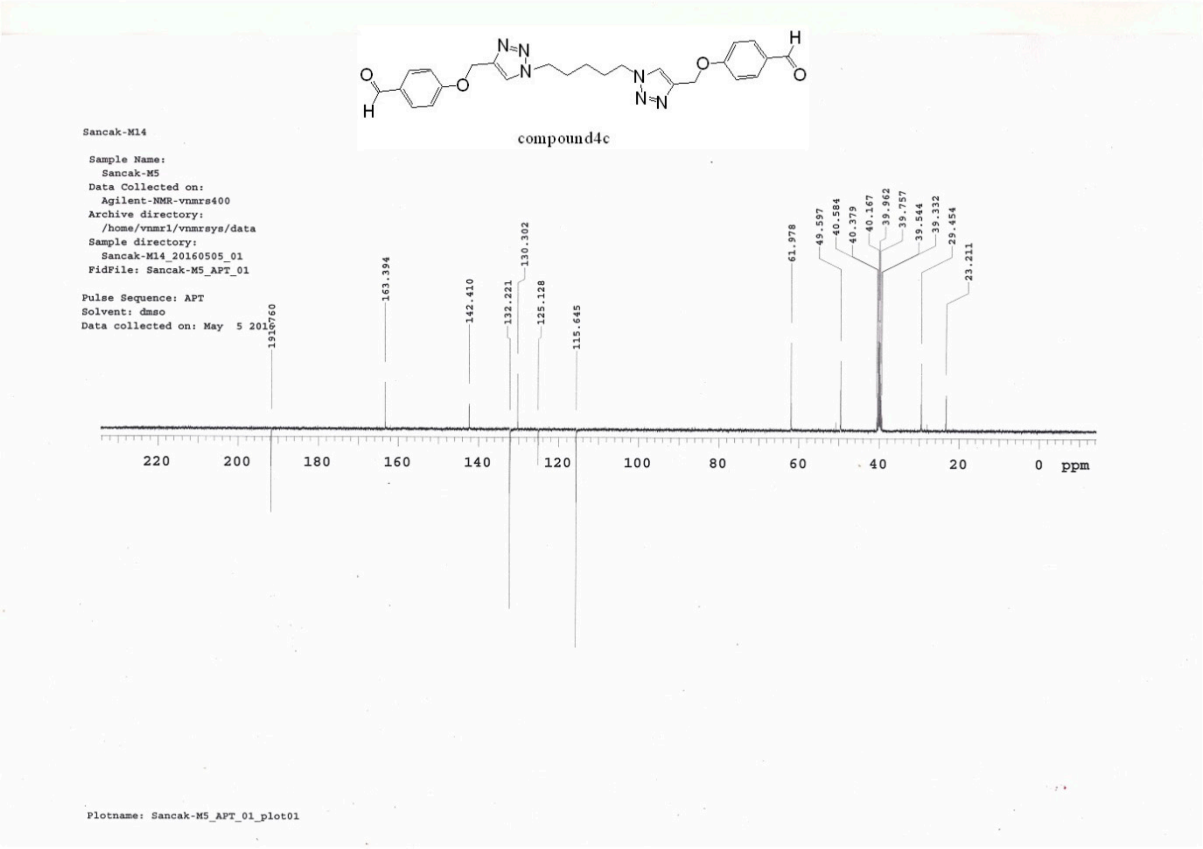

Figure S30. ^{13}C-NMR spectrum of compound **4c**.

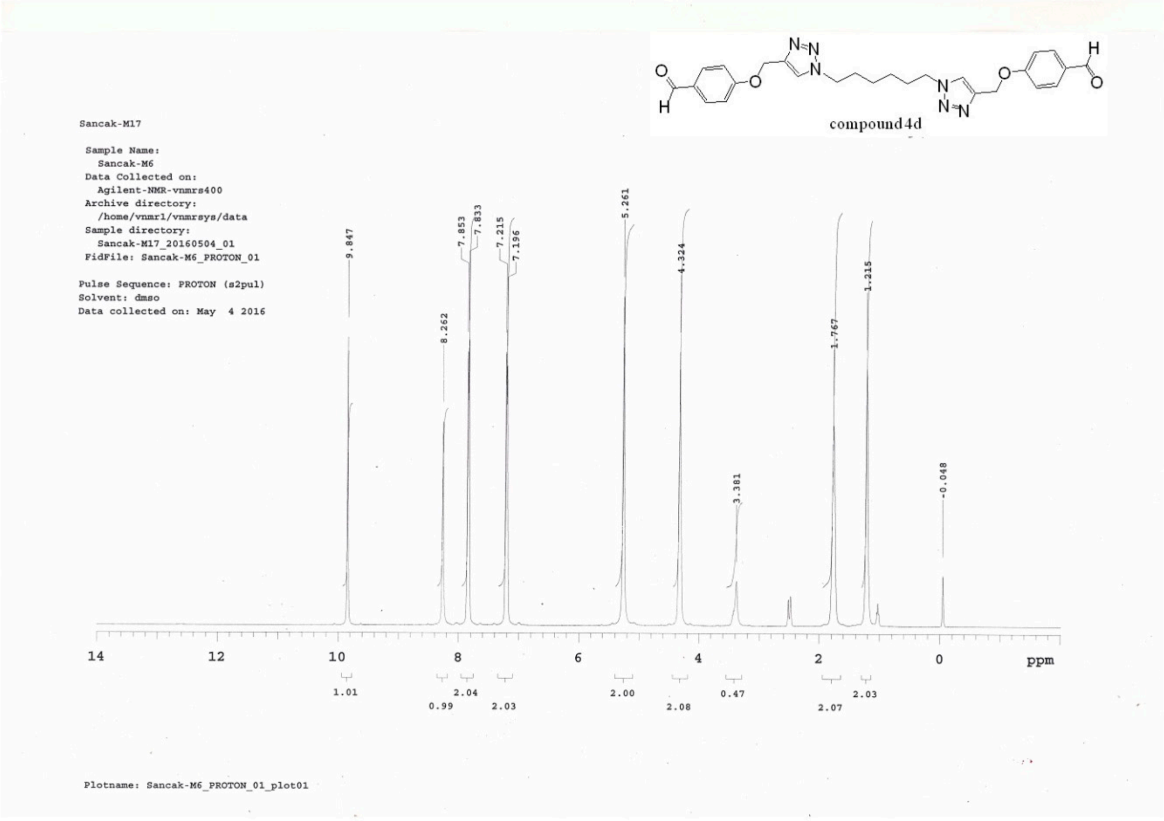

Figure S31. ^{1}H-NMR spectrum of compound **4d**.

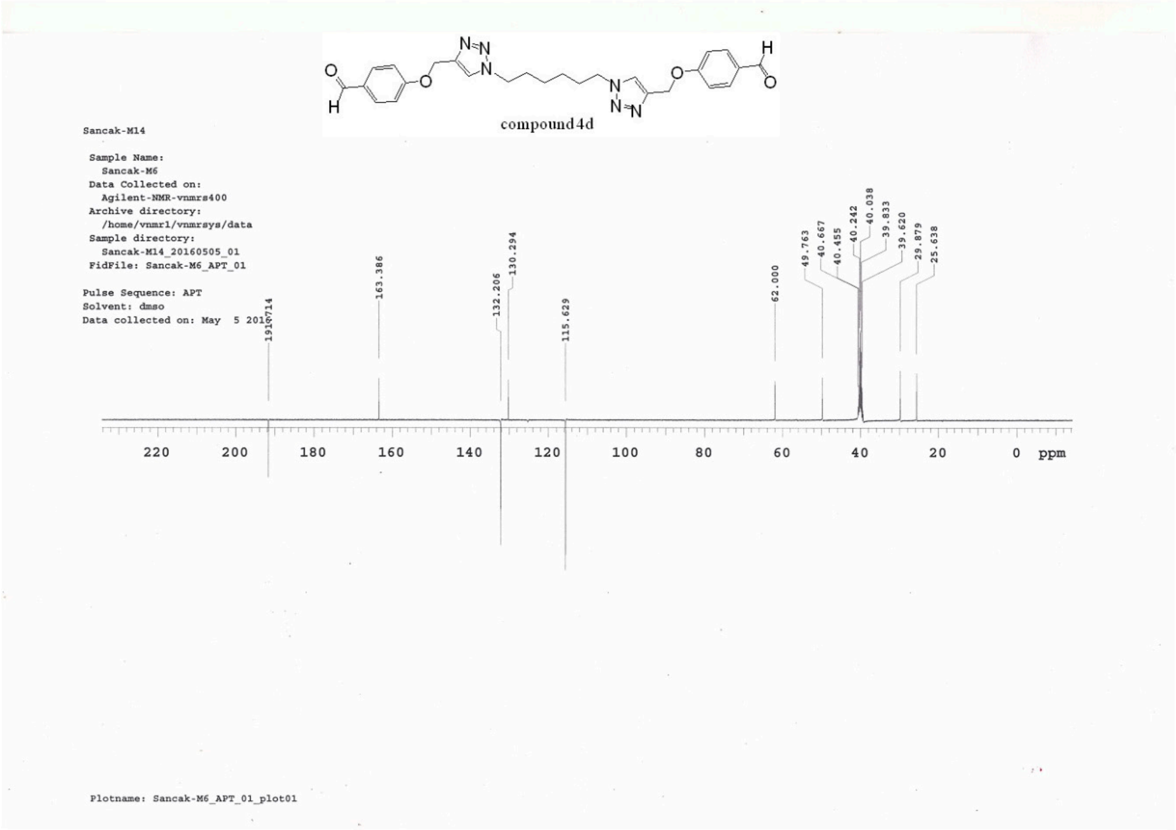

Figure S32. ^{13}C-NMR spectrum of compound **4d.**

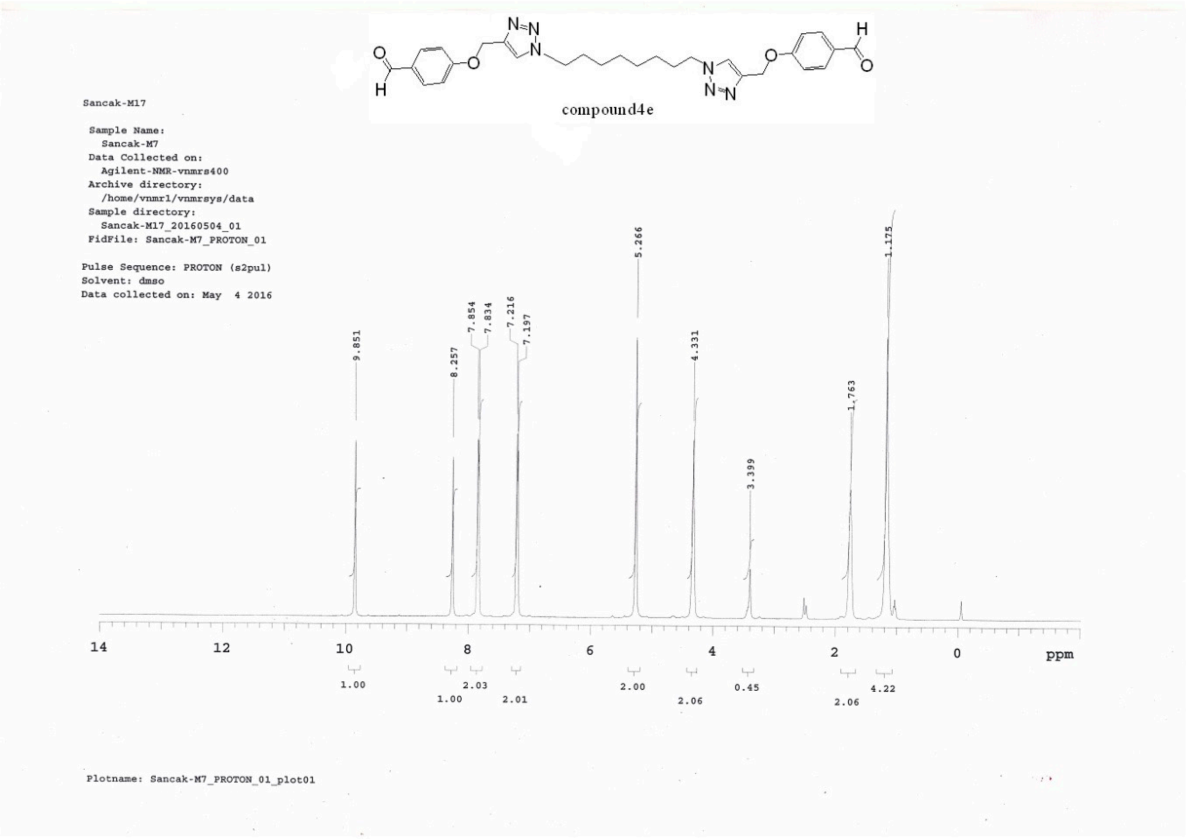

Figure S33. ^{1}H-NMR spectrum of compound **4e**.

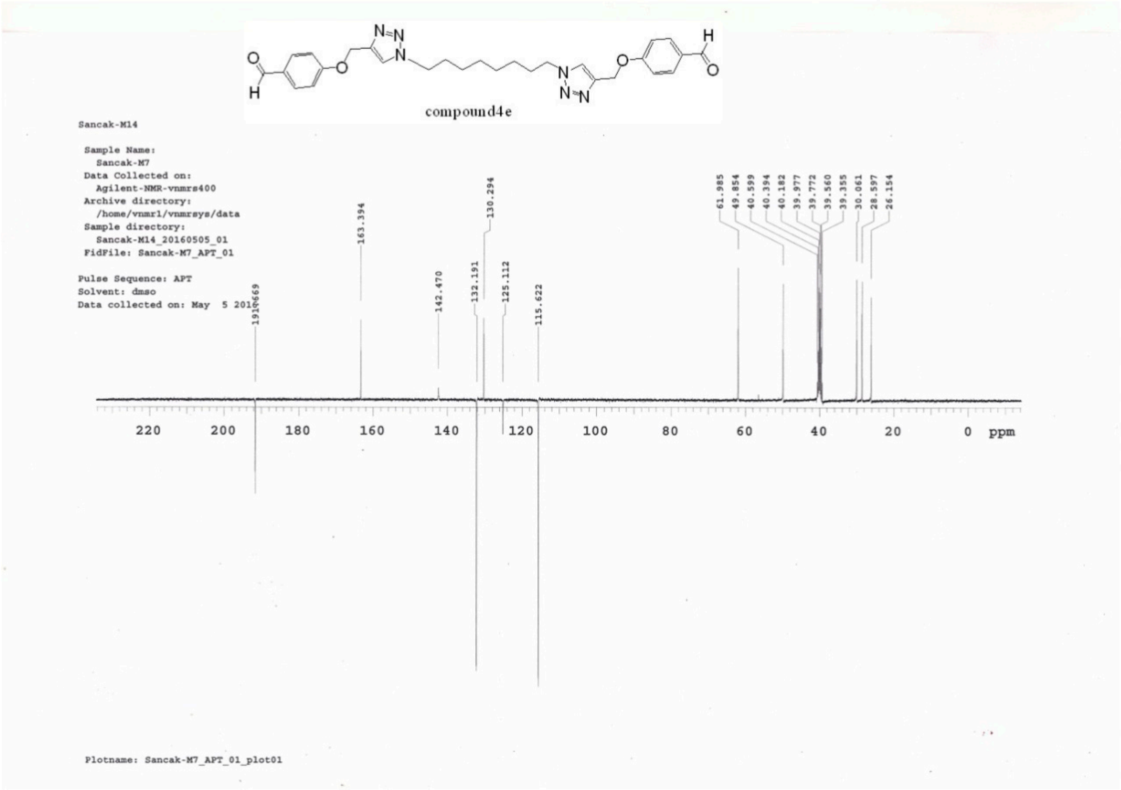

Figure S34. ^{13}C-NMR spectrum of compound **4e**.

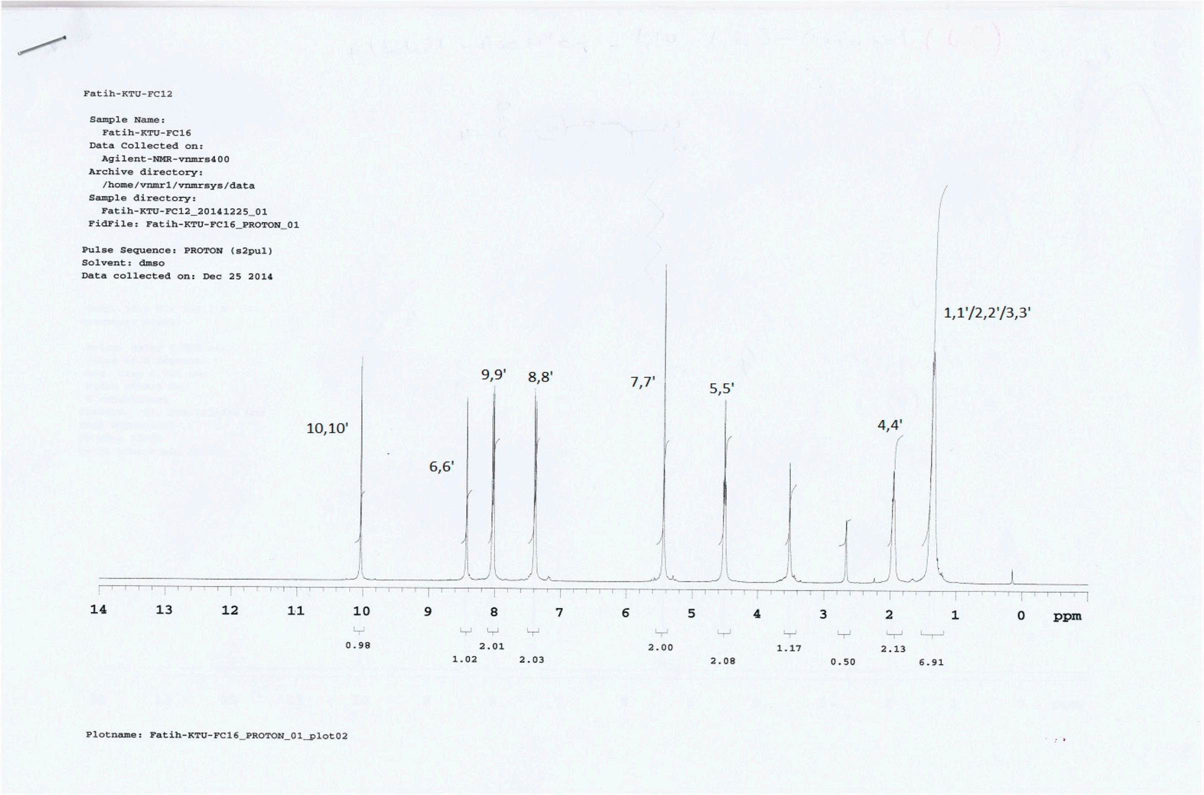

Figure S35. ^{1}H-NMR spectrum of compound **4f**.

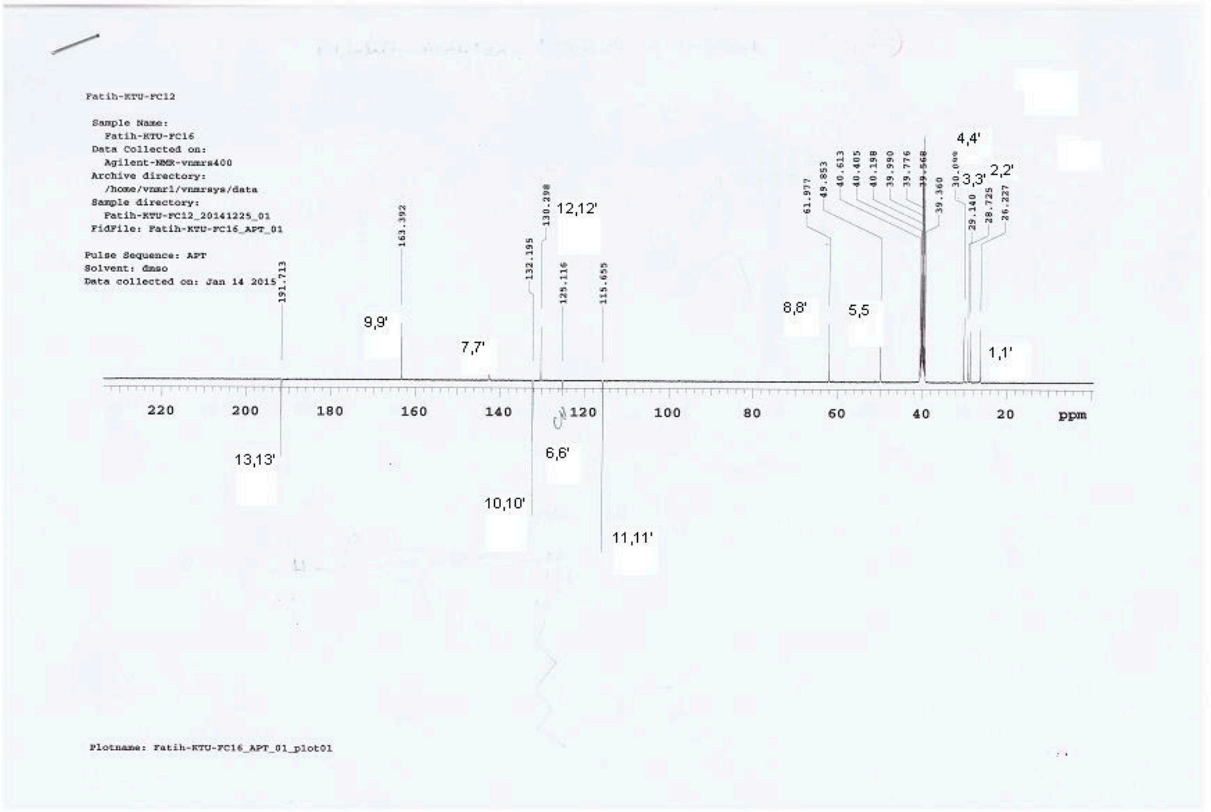

Figure S36. ^{13}C-NMR spectrum of compound **4f**.

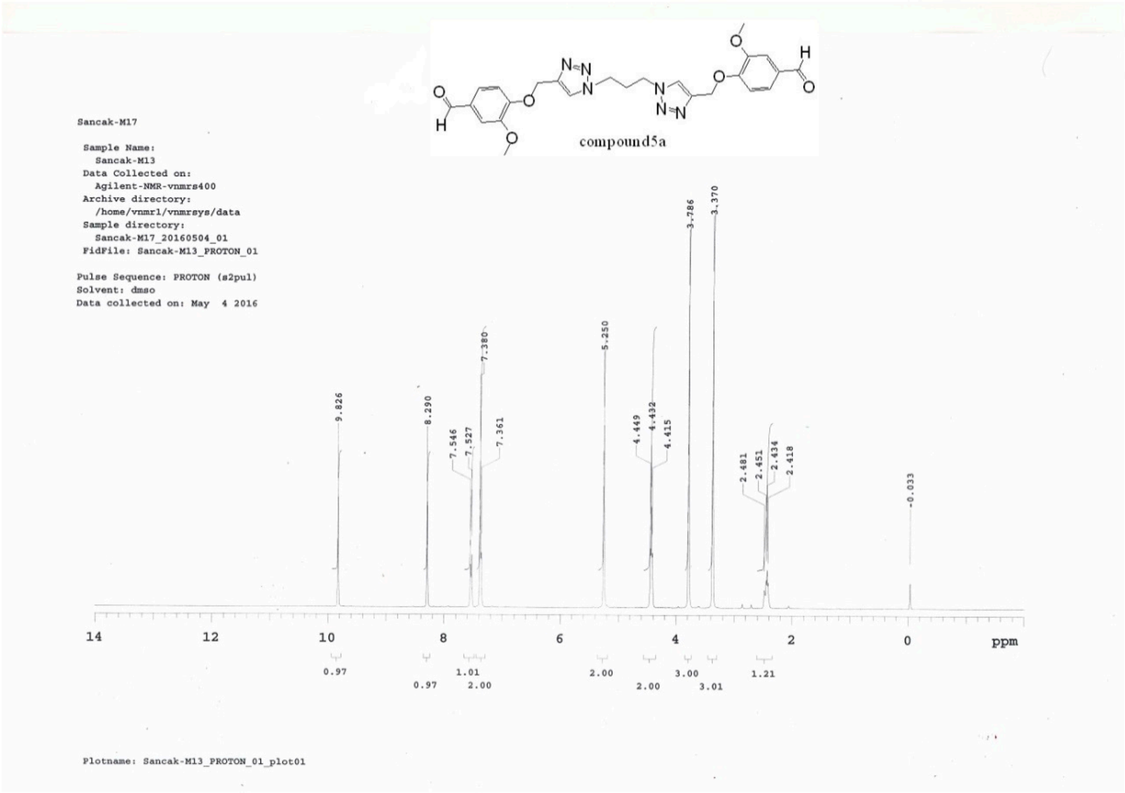

Figure S37. ^{1}H-NMR spectrum of compound **5a**.

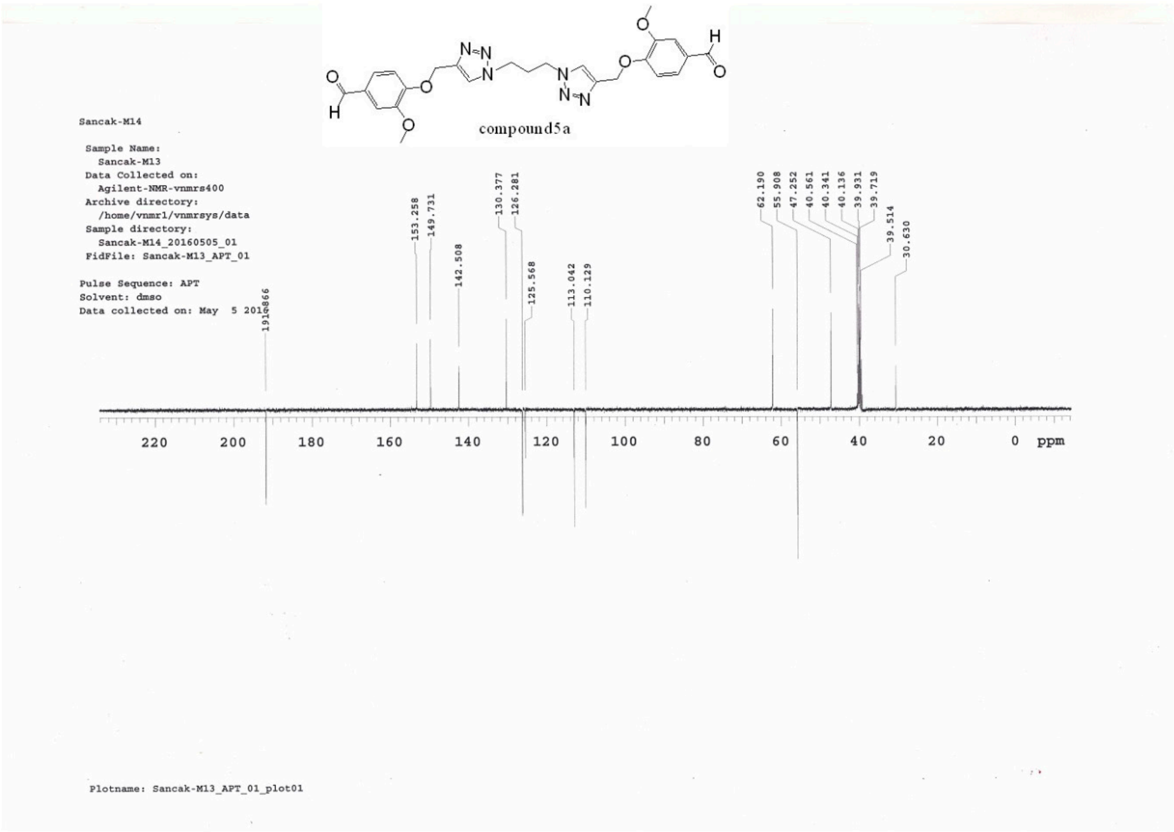

Figure S38. ^{13}C-NMR spectrum of compound **5a**.

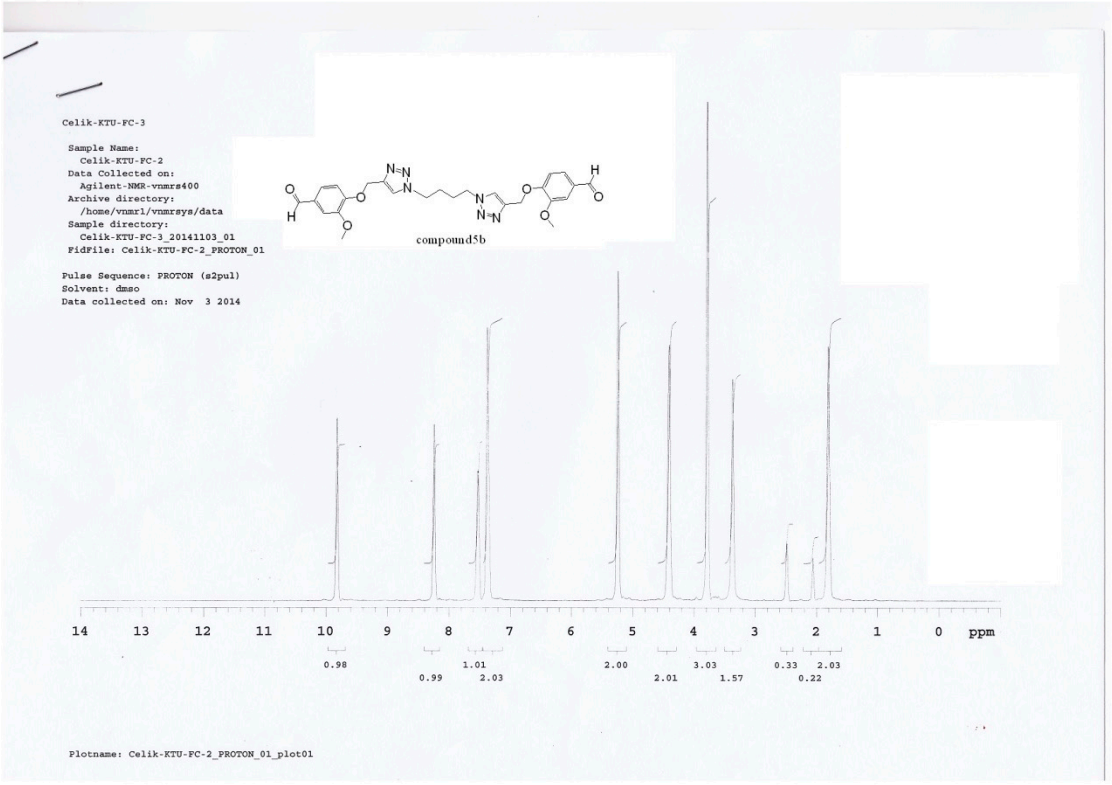

Figure S39. ^{1}H-NMR spectrum of compound **5b**.

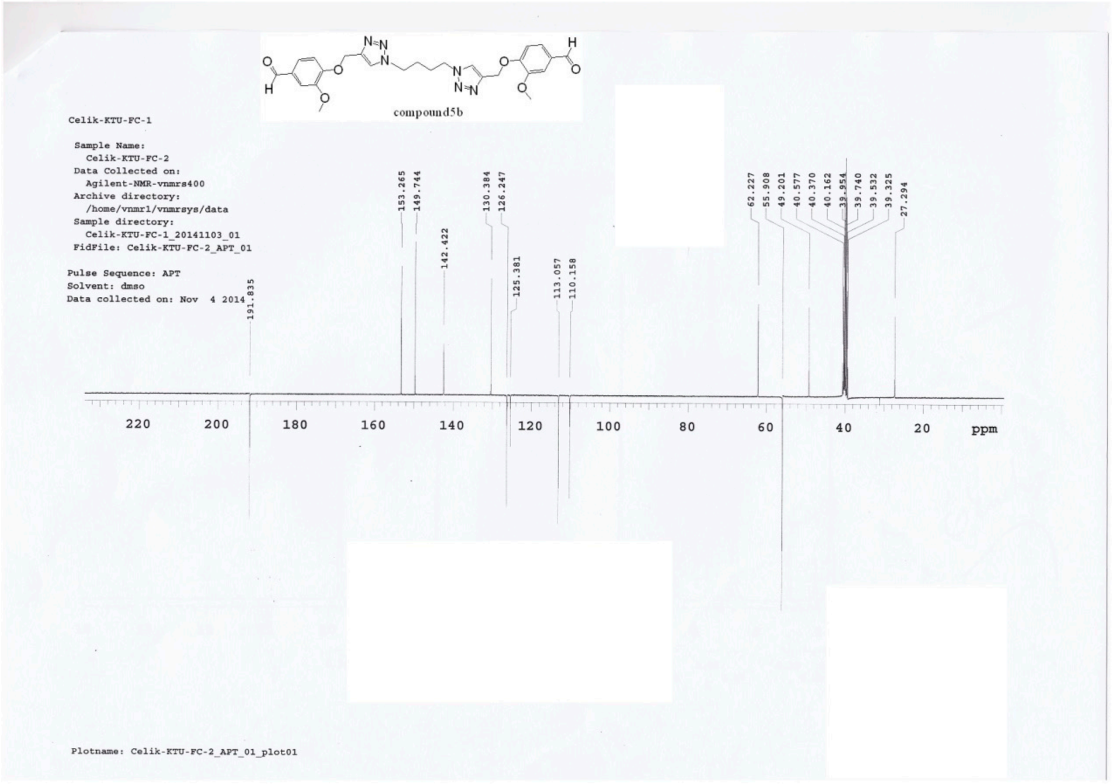

Figure S40. ^{13}C-NMR spectrum of compound **5b**.

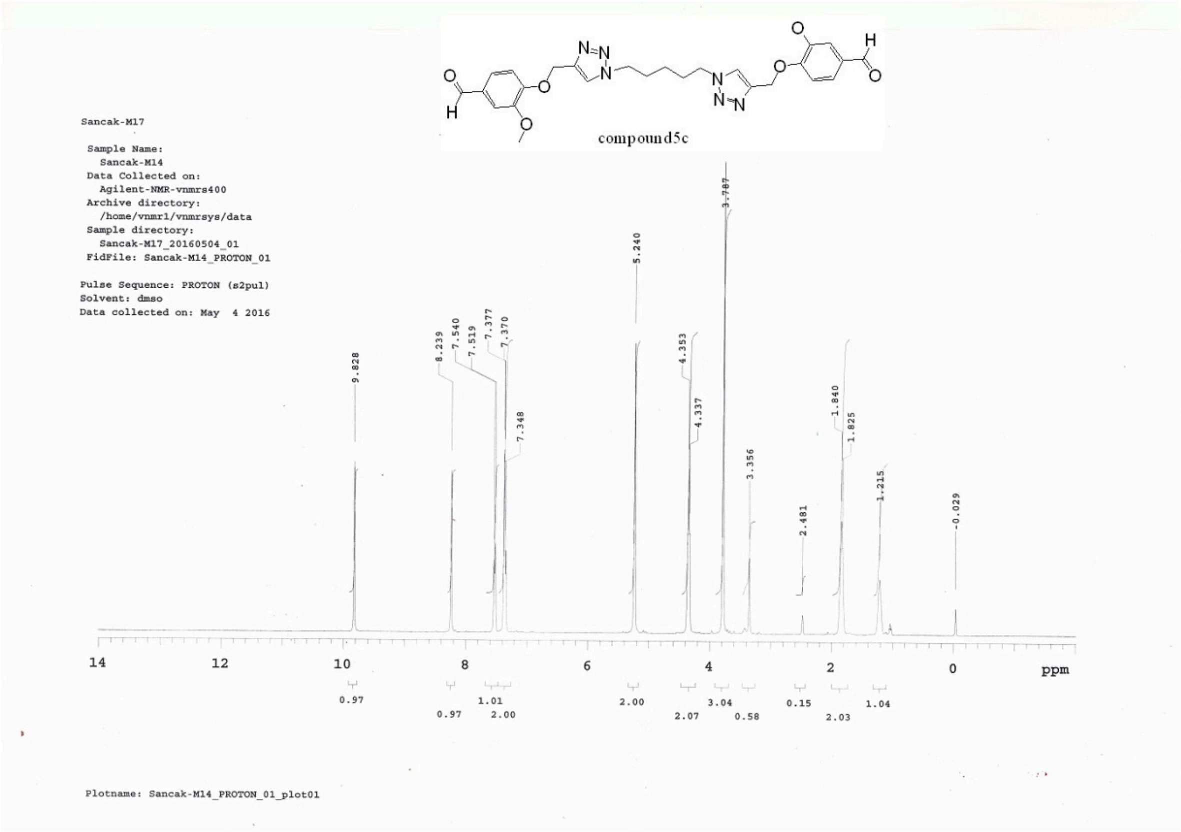

Figure S41. ^{1}H-NMR spectrum of compound **5c**.

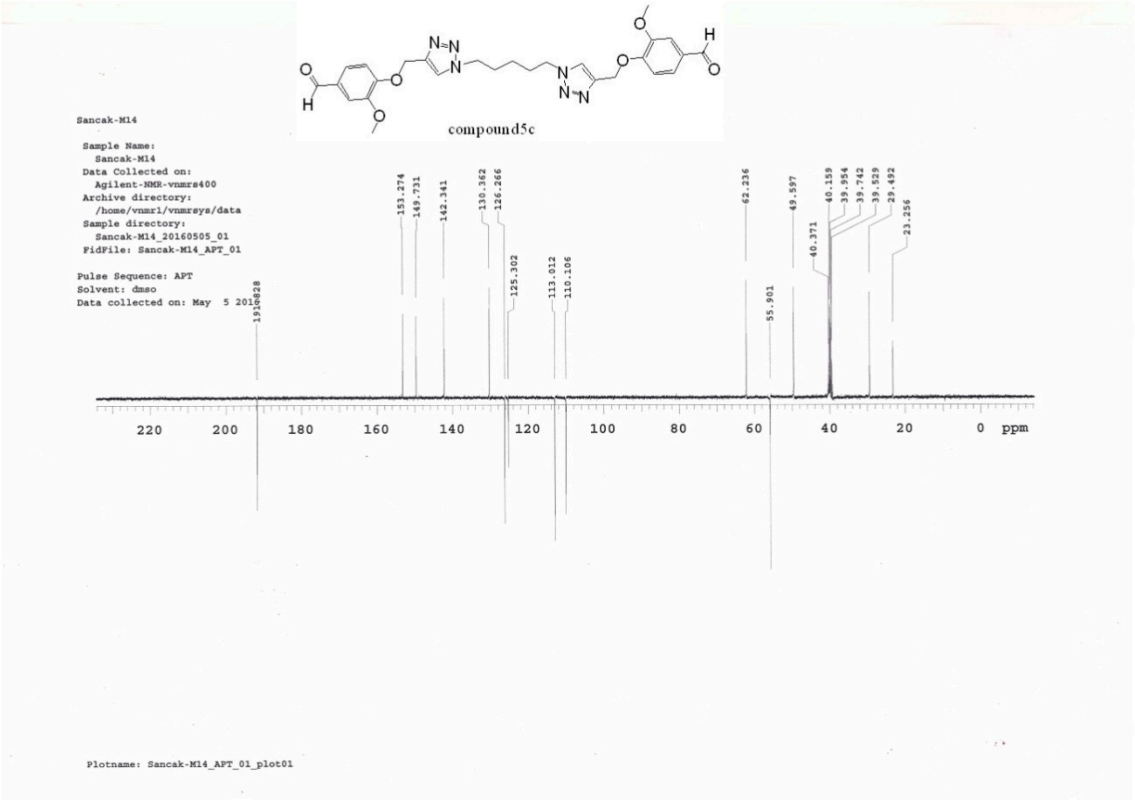

Figure S42. ^{13}C-NMR spectrum of compound **5c**.

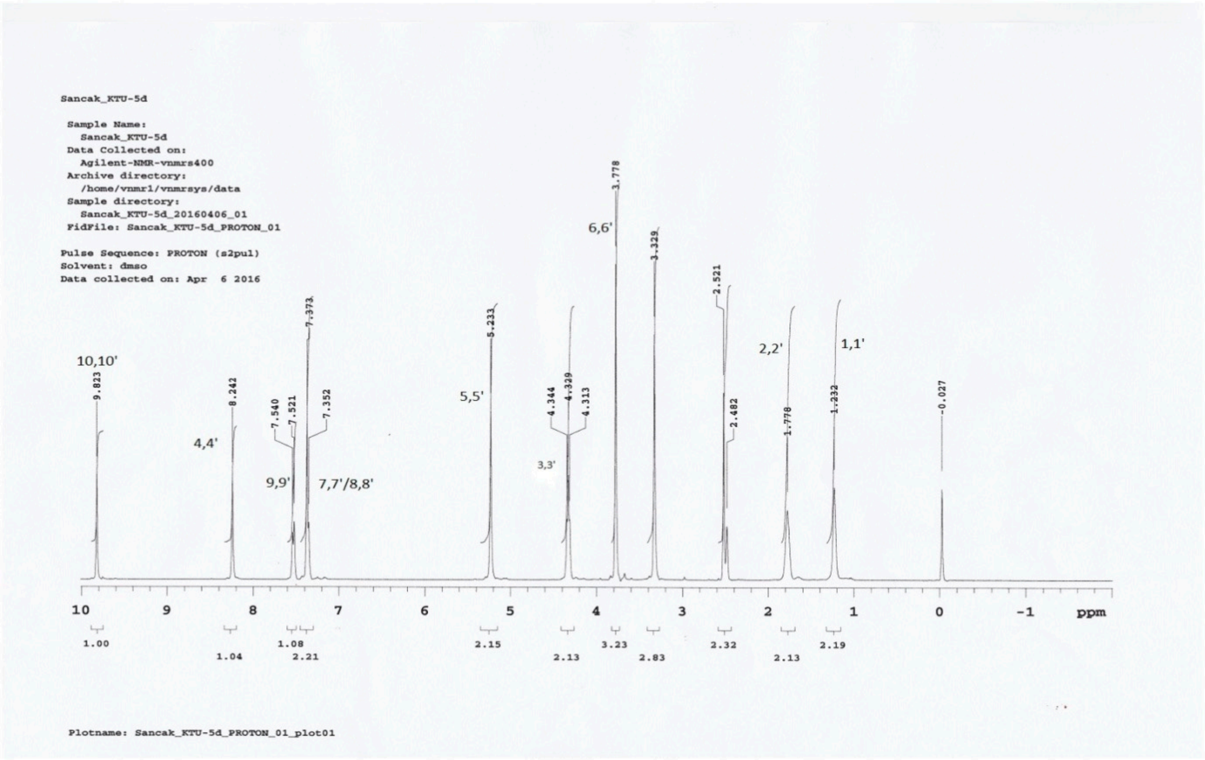

Figure S43. ^{1}H-NMR spectrum of compound **5d**.

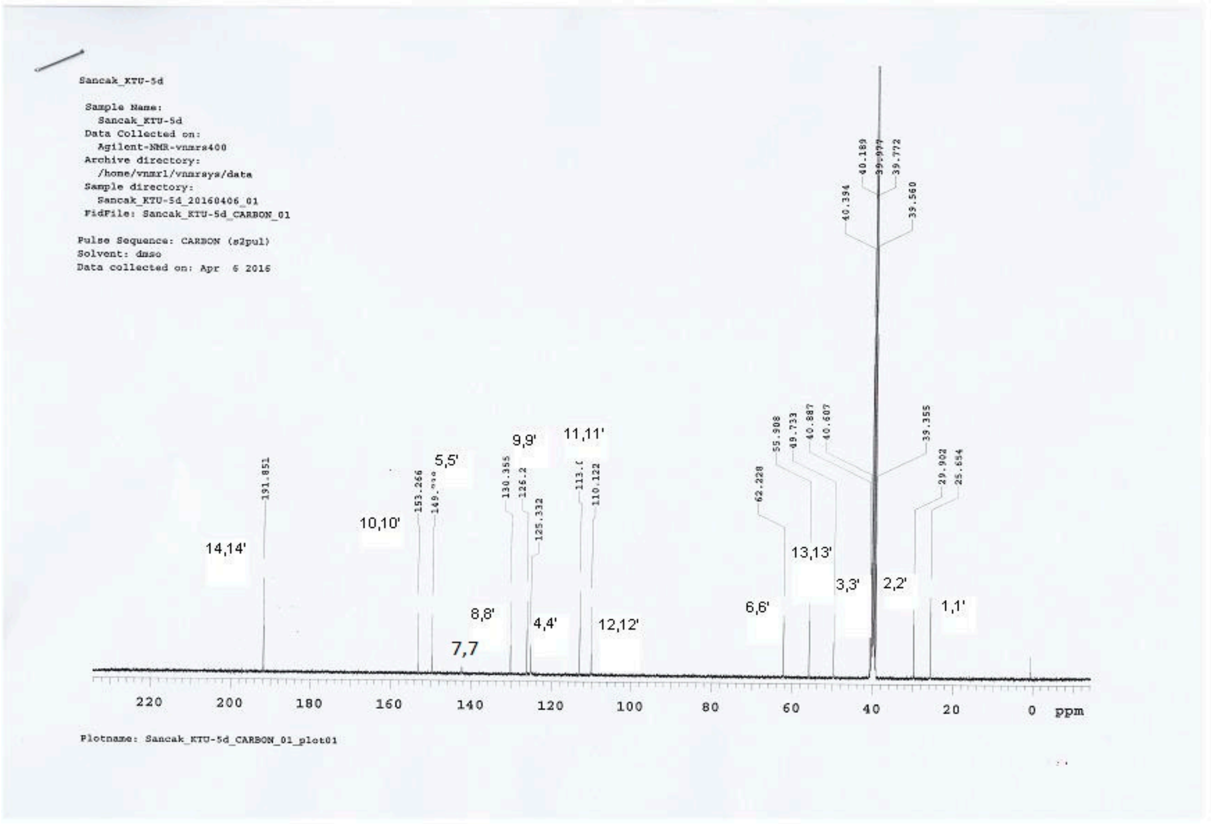

Figure S44. ^{13}C-NMR spectrum of compound **5d**.

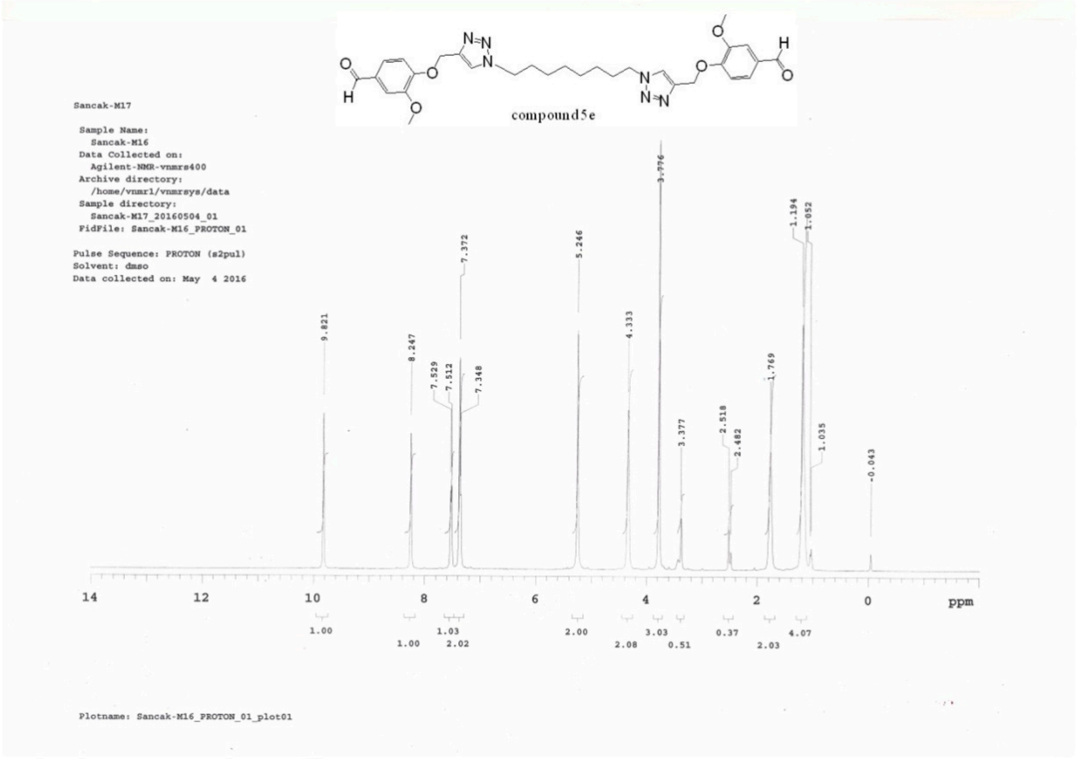

Figure S45. ^{1}H-NMR spectrum of compound **5e**.

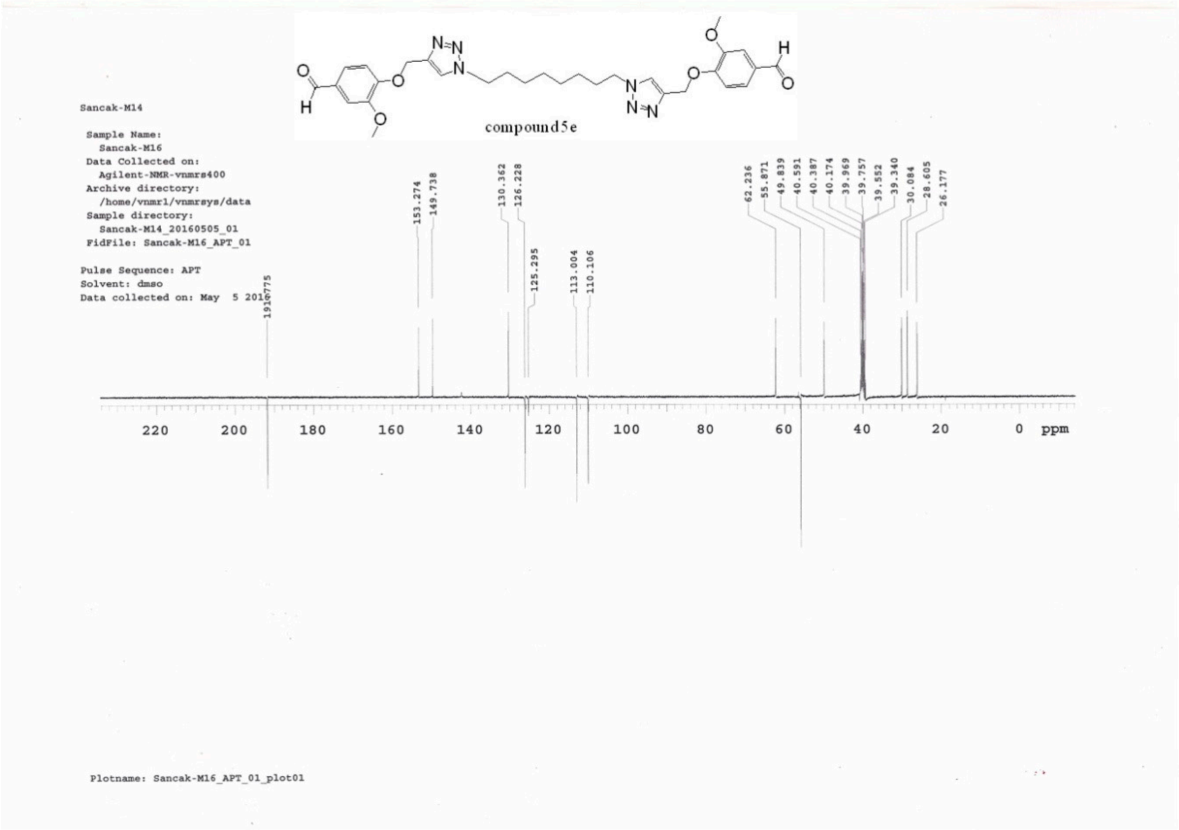

Figure S46. ^{13}C-NMR spectrum of compound **5e**.

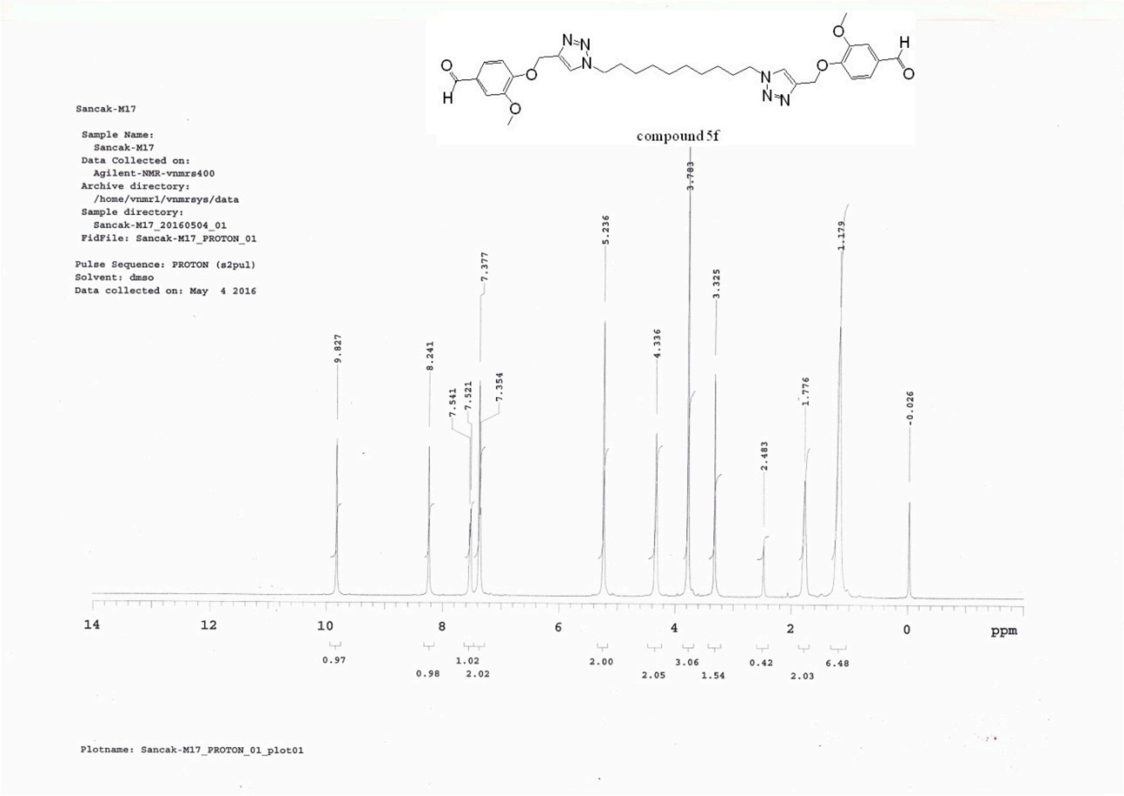

Figure S47. ^{1}H-NMR spectrum of compound **5f**.

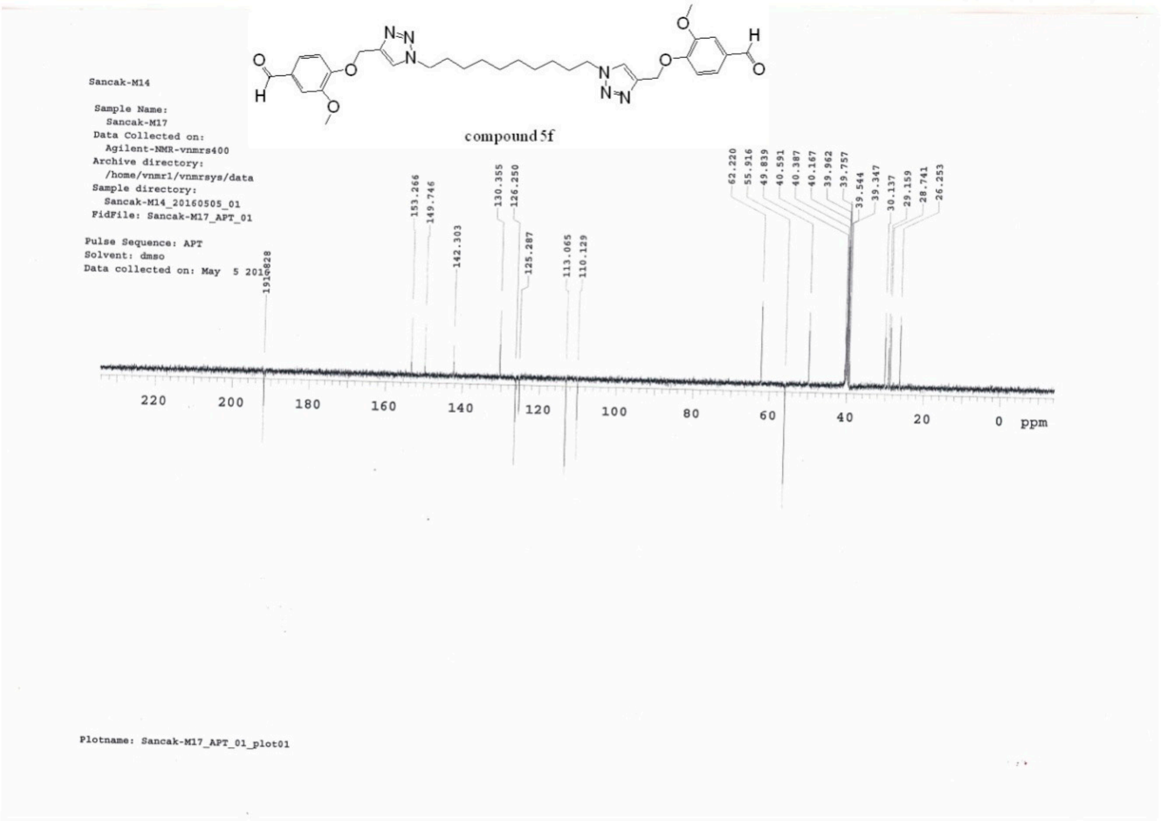

Figure S48. ^{13}C-NMR spectrum of compound **5f**.
